# Identification of a specific *APOE* transcript and functional elements associated with Alzheimer’s disease

**DOI:** 10.1186/s13024-024-00751-7

**Published:** 2024-08-29

**Authors:** Qiang Chen, Luis Aguirre, Guoming Liang, Huanhuan Zhao, Tao Dong, Felix Borrego, Itziar de Rojas, Qichan Hu, Christopher Reyes, Ling-Yan Su, Bao Zhang, James D. Lechleiter, Harald H. H. Göring, Philip L. De Jager, Joel E. Kleinman, Thomas M. Hyde, Pan P. Li, Agustín Ruiz, Daniel R. Weinberger, Sudha Seshadri, Liang Ma

**Affiliations:** 1 Lieber Institute for Brain Development, Johns Hopkins Medical Campus, Baltimore, MD USA; 2 Glenn Biggs Institute for Alzheimer’s & Neurodegenerative Diseases, University of Texas Health Science Center at San Antonio, 7703 Floyd Curl Dr, San Antonio, TX 78229 USA; 3 College of Animal Science, Shanxi Agricultural University, Taigu, Shanxi China; 4 Bioinformatics Program, University of Texas at El Paso, El Paso, TX USA; 5 Department of Psychiatry and Behavioral Sciences, Johns Hopkins University School of Medicine, Baltimore, MD USA; 6 Research Center and Memory Clinic, Ace Alzheimer Center Barcelona – Universitat Internacional de Catalunya, Barcelona, Spain; 7 Network Center for Biomedical Research in Neurodegenerative Diseases, National Institute of Health Carlos III, Madrid, Spain; 8 College of Food Science and Technology, Yunnan Agricultural University, Kunming, Yunnan China; 9 College of Forensic Medicine, Xi’an Jiaotong University Health Science Center, Xi’an, Shaanxi China; 10 Department of Cell Systems and Anatomy, University of Texas Health Science Center at San Antonio, San Antonio, TX USA; 11 South Texas Diabetes and Obesity Institute and Division of Human Genetics, University of Texas Rio Grande Valley School of Medicine, San Antonio, TX USA; 12 Center for Translational and Computational Neuroimmunology, Department of Neurology and Taub Institute for Research on Alzheimer’s Disease and the Aging Brain, Columbia University Medical Center, New York, NY USA; 13 Departments of Neurology, Neuroscience, and Genetic Medicine, Johns Hopkins University School of Medicine, Baltimore, MD USA; 14 Department of Neurology, University of Texas Health Science Center at San Antonio, San Antonio, TX USA; 15 Department of Neurology, Boston University School of Medicine, Boston, MA USA; 16 Department of Pharmacology, University of Texas Health Science Center at San Antonio, San Antonio, TX USA

**Keywords:** *APOE*, Alzheimer’s disease, Transcript, Postmortem brain, SNP

## Abstract

**Background:**

The *APOE* gene is the strongest genetic risk factor for late-onset Alzheimer’s Disease (LOAD). However, the gene regulatory mechanisms at this locus remain incompletely characterized.

**Methods:**

To identify novel AD-linked functional elements within the *APOE* locus, we integrated SNP variants with multi-omics data from human postmortem brains including 2,179 RNA-seq samples from 3 brain regions and two ancestries (European and African), 667 DNA methylation samples, and ChIP-seq samples. Additionally, we plotted the expression trajectory of *APOE* transcripts in human brains during development.

**Results:**

We identified an AD-linked *APOE* transcript (jxn1.2.2) particularly observed in the dorsolateral prefrontal cortex (DLPFC). The *APOE* jxn1.2.2 transcript is associated with brain neuropathological features, cognitive impairment, and the presence of the *APOE4* allele in DLPFC. We prioritized two independent functional SNPs (rs157580 and rs439401) significantly associated with jxn1.2.2 transcript abundance and DNA methylation levels. These SNPs are located within active chromatin regions and affect brain-related transcription factor-binding affinities. The two SNPs shared effects on the jxn1.2.2 transcript between European and African ethnic groups.

**Conclusion:**

The novel *APOE* functional elements provide potential therapeutic targets with mechanistic insight into the disease etiology.

**Supplementary Information:**

The online version contains supplementary material available at 10.1186/s13024-024-00751-7.

## Background

Alzheimer’s disease (AD) is a devastating neurodegenerative disease characterized pathologically by the accumulation of amyloid-β plaques and tau tangles, which leads to neuronal cell death and cognitive impairment. Most AD cases are non-Mendelian and late-onset (> 65 years old), and there is limited treatment available to slow down cognitive decline (e.g., lecanemab [1]), making AD the leading cause of mortality in the aging population [2]. African Americans remain underrepresented in AD research, despite the prevalence of AD possibly being double in frequency in African Americans compared to European Ancestry individuals [3].

The human APOE protein has three common isoforms defined by two single nucleotide polymorphisms (SNPs) that reside in the coding region of exon 4. Notably, the apolipoprotein E gene (*APOE*) epsilon 2 (*APOE2*) and epsilon 4 (*APOE4*) alleles are two major genetic risk factors for late-onset AD. Compared to the commonest genotype (homozygous genotype comprising two copies of the *APOE* epsilon 3, *APOE3/3*), people carrying two *APOE4* alleles (homozygotes) are at the highest risk [4]. Yet, there is no therapeutic intervention available to reduce this risk of *APOE4* carriers. Therefore, uncovering and understanding the biological effects regulating the expression of *APOE* isoforms might contribute to the control of this important AD risk factor.

Recently, we performed a genome-wide association study (GWAS) [5] and identified many AD-risk SNPs within the *APOE* gene region (Supplementary Fig. S1). However, most of these identified signals are in noncoding regions and are in complex linkage disequilibrium (LD) with other variants, including the SNPs encoding the protein isoforms of *APOE*. Although we suspect the existence of additional variants modulating the risk of *APOE* isoforms, the complexities within the locus might present difficulties in elucidating their potential modulation of AD-related risk alleles. *Cis*-acting expression quantitative trait loci (eQTLs) studies might help to improve our understanding of the mechanisms of AD-associated variants in the regulation of the *APOE* gene expression [6, 7]. Interestingly, a splicing variant of *APOE* mRNA with intron-3 retention, a long noncoding RNA, was found to govern *APOE* gene expression in neurons [8]. Furthermore, this noncoding RNA of *APOE* is more abundant in AD patients with more severe tau and amyloid pathological burden [9]. In contrast, the role of each *APOE* protein-coding transcript in AD pathogenesis is still unclear. A study between *APOE* transcription and AD pathology has been attempted in AD brains from the superior temporal gyrus, but no significant correlation was determined [10].

Another challenge is to understand the specific mechanism(s) by which variations at the *APOE* locus alter risk, including DNA methylation, chromatin activity, transcription factor (TF) binding, and their interactions with SNPs and specific *APOE* transcripts. Changes in the level of DNA methylation in brain tissue were observed in AD subjects in the *APOE* CpG islands within exon 4 compared to age-matched controls [11]. Chip-seq of histone marks has been generated at the *APOE* locus from several studies [12]. However, how common risk alleles influence the epigenetic elements in AD remains largely unknown.

The present study aimed to connect common AD risk alleles at the *APOE* locus with transcript(s), CpGs, and active chromatin regions by combining available human postmortem brain high-throughput functional genomics data. We leveraged two large human autopsy brain cohorts collected by the Religious Orders Study/Memory and Aging Project (ROSMAP) [13] and the Lieber Institute for Brain Development (LIBD) [14]. Overall, we deepen our understanding of the genetic and epigenetic regulation of *APOE* in the postmortem brain and provide a foundation for formulating mechanistic hypotheses for the variants within APOE associated with AD risk.

## Methods

### ROSMAP

#### WGS data processing

Whole-genome sequencing (WGS) datasets were collected by the ROSMAP consortium [13]. There were 43,012,378 genomic variants in the raw data. Genetic variants were filtered out with PLINK 1.9 [15] if they: (1) had more than two alleles; (2) had a genotype missing rate > 10%; (3) had Minor Allele Frequencies (MAF) < 1%; and (4) deviated from Hardy–Weinberg Equilibrium (HWE, *p-value* < 1E − 6). Finally, we retained 9,912,554 common SNPs (23% of the total genetic variants).

### IBD and PCA

To detect genetically related samples and population stratification, we applied stricter Quality Control (QC) procedures before conducting the Identity-By-Decent (IBD) test and Principal Component Analysis (PCA). First, we merged the study data with HapMap3 data and kept only the overlapped SNPs. We then removed SNPs if they: (1) had a genotype missing rate > 1%; (2) had MAF < 5%; (3) deviated from HWE (*p*-value < 1E − 3), and (4) were in Major Histocompatibility Complex (MHC) regions (chr6:25 M-33.5 M). Finally, we retained 995,871 variants for further analysis. Pruning was conducted twice using PLINK with option –indep- pairwise 200 100 0.2. IBD test was conducted using PLINK with option –genome. Subjects with PI-HAT > 0.2 were identified as the related subjects, and one of the related subjects with a higher overall SNP missing rate of the pair was removed. PCA was conducted with EIGENSOFT 6.1.3 [16]. Twenty PCs were kept. Outliers of the population were detected in a training-prediction approach. We classified HapMap3 samples into two groups: EUR (CEU, TSI) and others. Next, we used 20 PCs of HapMap samples to fit a general linear model with glmnet, and then we used an estimated model to predict the probability of ancestry (ancestry score) for the studying sample. Subjects with ancestry scores lower than 0.8 were removed from study samples.

#### Bulk brain RNA-Seq data processing

Three brain regions of postmortem data were included in this study. The details of sample information can be found in Table 1 and Supplementary Table S1. The protocol of sample procurement has been described previously [13, 17]. QC of the sequence data, including checks for over-abundance of adaptors and over-represented sequence, was performed using FastQC. Low-quality reads (5% of the total) were filtered out using the Trimmomatic [18], which is a fast, multithreaded command line tool to trim and crop FASTQ data and remove adapters [18]. After trimming adapter sequences, reads passing initial QC were aligned to the human reference genome using HISAT2 [19]. Gene lengths were obtained from GENCODE v41 annotations [20]. We normalized gene counts to Reads Per Kilobase per Million mapped reads (RPKM) values and junction counts to Reads per 10 Million (RP10M) values using the total number of aligned reads across the 22 autosomal chromosomes. Normalized values can be interpreted as the number of reads supporting the junction in average library size [21].

**Table 1:** Sample information on bulk brain tissue

**Brain collection**	**Assay**	**Ancestry**	**Brain region**	**Sample size**	**Diagnosis**	**Gender (male/female)**	**Age range (mean/sd)**
**ROSMAP**	RNA-Seq	European	AC	433	AD/NC: 271/162	269/164	70.64-90.00 (87.03/4.19)
		European	DLPFC	573	AD/NC: 365/208	372/201	70.27-90.00 (86.71/4.50)
		European	PCC	499	AD/NC: 312/187	309/190	70.64-90.00 (86.97/4.22)
	ChIP-Seq	European	DLPFC	615	AD/NC: 399/216	398/217	65.99-90.00 (86.49/4.57)
	Methylation	European	DLPFC	667	AD/NC: 426/241	424/243	65.99-90.00 (86.34/4.68)
**LIBD**	RNA-Seq	European	DLPFC	376	SCZ/BIP/MDD/NC: 93/54/125/104	246/130	13.02-96.92 (43.62/15.67)
		African	DLPFC	216	SCZ/BIP/MDD/NC: 76/6/13/121	135/81	13.00-85.14 (45.75/16.09)

Brain collection: *ROSMAP* The Religious Orders Study (ROS) and the Memory and Aging Project (MAP), *LIBD* Lieber Institute for Brain Development. Brain region: *DLPFC* dorsolateral prefrontal cortex, *PCC* posterior cingulate cortex, *AC* anterior cingulate cortex. Diagnosis: *AD* Alzheimer’s disease based on CERAD score, *NC* normal control, *SCZ* schizophrenia, *BIP* bipolar disorder

#### eQTL analysis

eQTL association was examined separately by feature type (gene and junction) using TensorQTL package [22], taking log2-transformed expression levels of each measurement (RPKM and RP10M) as the input. Features with low expression (average counts < 0.4 in gene and < 0.1 in junction) were excluded before eQTL analysis. To control for potential confounding factors, we adjusted expression levels using first 5 PCs from the genotype data, diagnosis, sex, age, RIN, rRNA rate, and the first K PCs of the log2-transformed expression levels, where K was calculated separately by feature type using the sva Bioconductor package [23]. Log2-transformed expression data, SNP genotype data, and all covariates were taken as inputs for TensorQTL cis.map_cis function to fit a general linear model as shown in Model-1 in Supplementary Table S2. False discovery rate (FDR) was assessed across all *cis*-eQTL tests within *APOE* region using R package qvalue [24]. We considered all variant–gene pairs (expression features to genes, eGene) and variant–junction pairs (eJunction) when the distance between features and SNP is < 1 MB.

#### Conditional analysis on *APOE*2&4 genotypes

We evaluated the effects of *APOE* loci on associations of candidate SNPs with the expression of *APOE* gene and its transcripts. To evaluate the effect of *APOE2,3,4* genotypes on our association, we first generated a variable *APOE4* (4 carriers and non-4 carriers) and a variable *APOE2* (2 carriers and non-2 carriers). We used the ROSMAP-provided *APOE2,3,4* genotypes to evaluate their conditional effect. Since we don’t have data for *APOE* genotypes in the LIBD sample, we used two *APOE2,3,4-*determining SNPs (rs7412 and rs429358) to derive the *APOE* genotypes according to Supplementary Table S3. We then fitted *APOE4* and *APOE2* to Model-2 in Supplementary Table S2 to examine the association of our candidate SNPs and *APOE* expression. If the *p*-value of likelihood ratio test is larger than 0.05, we concluded that the effect of candidate SNPs is independent of *APOE* genotype.

#### Epistasis of candidate SNPs and *APOE*2/4 genotypes on expression of *APOE* transcripts

We used likelihood ratio test to examine the difference of variance explained between model including *APOE4* and *APOE2,* and the model without *APOE4* and *APOE2* (see Model-3 in Supplementary Table S2). If the *p*-value of likelihood ratio is larger than 0.05, we concluded that there is no interaction between candidate SNP and *APOE* genotypes on response variables.

#### Differential expression analysis

We used a general linear Model-4 in Supplementary Table S2 to investigate the differential expression of *APOE* gene and transcripts in 5 different diagnosis groups. We first fit a general linear model using Sex, Age, RIN, rRNA-Rate, the total number of assigned genes, 5 SNP PCs, and K number gene PCs used in eQTL analysis to keep consistency. We took the residual as the adjusted expression levels for further examination. Using the adjusted expressions, we conducted an ANOVA test using Anova in R to evaluate the difference between diagnosis groups. We also used the adjusted expressions for the related plots.

#### DNA methylation data processing

Methylation data from brain DLPFC of 743 individuals were collected using the Illumina HumanMethylation450 BeadChip by the ROSMAP consortium. After matching to QCed genotype data, we got 667 samples. QC and normalization were conducted using minfi R package [25]. Failed positions were identified with detectionP function in minfi by examining both the methylated and unmethylated channel reporting background signal levels. *P*-value for every genomic position in every sample was estimated. Small *p*-values indicate a good position. We excluded samples with averaged *p*-values > 0.05 across all probes, and also removed probes with averaged *p*-values > 0.05 across all samples. Normalization was conducted with function preprocessQuantile. We excluded probes on sex chromosomes to focus on mQTLs analysis on autosome chromosomes. We also removed probes that have the same locations as SNPs.

#### mQTL analysis

*cis*-mQTL association was examined for CpG using TensorQTL package [22]. To control for potential confounding factors, we included co-variants: sex, age, and diagnosis. As shown in Model 5 in Supplementary Table S2, we also included the first five PCs from the genotype data to adjust population stratification, and the first 2 Negative control PCs to adjust potential batch effect. The number of negative control PCs was calculated with R Bioconductor package sva [26] using QCed methylation data. FDR was assessed in R package qvalue [24] across all QTL tests in the *APOE* region. We considered all variant–CpG pairs when the distance between CpG and SNP is < 1 MB.

#### ChIP-Seq data processing

Trim Galore was used to check the quality of the FASTQ files and run trimming. Bowtie 2 was used to align FASTQ files while the output was converted to the SAM file format. Samtools view was used to convert SAM files to BAM format. Bedtools intersect function was used to remove chrM, chrUN, pcr dup done with parameters, where blacklist is a list of unwanted sequences from the human reference genome. This output was then sorted using Samtools sort and potential PCR duplicates were removed using Samtools rmdup. To create bigWig file formats, deepTools bamCoverage was used for ChIP-seq peaks visualization via the WashU genome browser. To obtain DNA binding motifs, we used Motif Scan and Enrichment Analysis (MoSEA) to scan for motifs. MoSEA can search for motifs against specified position weight matrices (PWMs). We used the HOmo sapiens COmprehensive MOdel COllection (HOCOMOCO) v11 mononucleotide in MEME format as the PWMs. MoSEA also incorporates MEME Suite’s Find Individual Motif Occurrences (FIMO) [27] tool to scan for sets of sequences for individual matches to all motifs in HOCOMOCO v11 [28].

### LIBD

#### Genotype data processing

SNP genotyping with HumanHap650Y_V3, Human 1 M-Duo_V3, and Omni5 BeadChips (Illumina, San Diego, CA) was conducted with DNA extracted from brain cerebellar tissue [21]. Genotype imputation was performed on TOPMed server with the imputation reference from the Human Reference Forum (https://topmedimpute.readthedocs.io/en/latest/). We retained common SNPs (MAF > 5%) that were present in the majority of samples (missingness < 5%) that were in HWE (*p*-value > 1 × 10^−6^) using the PLINK 1.9 [15]. 9,984,191 SNPs were retained after QC.

### IBD and PCA

Further QC procedures were conducted for IBD and PCA using the same pipeline as those for ROSMAP data. After QCs, 847,380 variants with LIBD and HapMap3 data in common were retained for analysis. We conducted the same procedure to detect related samples and created 20 PCs using EIGEINSOFT 6.1.3 [16]. Outliers were removed following the same pipeline as present in ROSMAP data. European Ancestry and African Ancestry were separated in eQTL analysis. The first 5 PCs were used for correcting population stratification along with other covariates in eQTL analysis.

#### Bulk brain RNA-Seq data processing

DLPFC RNA-Seq data from postmortem brain samples were included in this study. Details of tissue acquisition, handling, processing, dissection, clinical characterization, diagnoses, neuropathological examinations, RNA extraction, and quality control measures were described previously [29]. RNA extraction, sequencing, and RNA data processing were also described previously [21]. In our analysis, gene lengths were calculated using GENCODE v41 annotations [20]. We normalized gene counts and junction counts using the same approach as we did for ROSMAP data.

#### eQTLs analysis

eQTL association was examined separately by ancestry (European and African) using TensorQTL package [22], taking log2-transformed expression levels of each measurement (RPKM and RP10M) as the input. Features with low expression (average counts < 0.4 in gene and < 0.1 in junction) were excluded before eQTL analysis. We used the same Model-1 (Supplementary Table S2) as ROSMAP for eQTL analysis with different expression PCs estimated by the sva Bioconductor package [23].

#### Differential expression analysis

We used the same general linear Model-4 (Supplementary Table S2) as ROSMAP to investigate the differential expression in diagnosis groups, including schizophrenia, bipolar disorders, major depression disorders, and controls.

#### BrainSpan RNA-seq data

BrainSpan is a consortium for studying transcriptional mechanisms involved in human brain development. BrainSpan has 42 samples from 21 brain regions. These samples include 19 fetus and 23 child/adult brain tissues from 0 to 40 years old. We used the same pipeline as ROSMAP to process a total of 558 RNA-seq data. Gene and Junction counts were estimated. After screening, 16 brain regions (sample size > 5) and 41 samples with 50% and more brain regions were included in the study (Supplementary Fig. S2). PCA was conducted using 41 samples by 16 brain regions count matrix, and plot using PC1 was created for evaluating the developmental trajectory of RNA-seq expression in fetus, child, and adult human brain.

#### Local ancestry

Local ancestry was estimated using RFMIX package [30], which uses random forest machine learning methods combined with a conditional random field model to identify the ancestry of genomic segments. We used 1000 genome phase 3 data as reference. All samples of 1000 genome were classified into 5 super populations (AFR: Africans; AMR: Admixed Americans; EAS: East Asians; EUR: Europeans; SAS: South Asians). ROSMAP and LIBD genotype data were phase-resolved using Eagle/conform program. Since we focused on *APOE* region in this study, we ran RFMIX software using genetic data in *APOE* region extended 1 M base pairs. From the RFMIX estimation, we used EUR or AFR score instead of whole genome PCs as population stratification covariate in TensorQTL analysis for European or African samples, as shown in Model-6 in Supplementary Table S2.

### SMR

Summarized-data-based Mendelian Randomization (SMR) uses summary-level statistics from GWAS and eQTL to test pleiotropic association between the expression levels of a gene and a complex trait [31]. We used eQTLs results with *p* < 0.05 from each brain region and summary stats of AD GWAS with *p* < 0.0001 as inputs, and 1000 genome as reference to conduct SMR test.

## Results

**Fig. 1:**
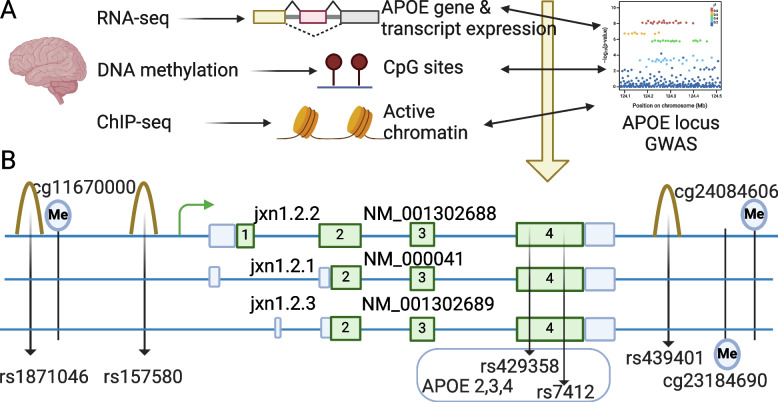
Overview of *APOE* study in human postmortem brain (**A**) and novel AD risk factors (genetic, transcriptomic, and epigenetic elements) and their relative position at the *APOE* locus (**B**). Brain collection: ROSMAP, The Religious Orders Study and the Memory and Aging Project; LIBD, Lieber Institute for Brain Development. Ancestry: EA, European Ancestry; AA, African American. Brain region: DLPFC, dorsolateral prefrontal cortex; PCC, posterior cingulate cortex; AC, anterior cingulate cortex. Green boxes represent exons; blue boxes represent untranslated regions (UTRs); Me represents methylation site; peaks represent active chromatin regions; 3 solid blue lines represent genomic DNA

To elucidate the mechanism of AD risk variants and its connections with transcriptomic, genetic, and epigenetic features within the context of AD, we harnessed the power of available multi-omics datasets sourced from diverse brain regions and two ancestries. Table 1 contains comprehensive demographic information pertaining to the participants in our analysis. It is noteworthy that while certain facets of this dataset have previously been analyzed in studies exploring brain phenotypes [21, 32], these earlier investigations predominantly emphasized genome-wide patterns. In contrast, our current study is distinct in its focus to unravel the intricate regulatory mechanisms operating within the *APOE* locus. As Fig. 1A and Supplementary Fig. S3 illustrate, we link AD genome-wide significant risk alleles (‘AD alleles’ hereafter) at the *APOE* locus to *APOE* gene and transcripts expression. Then, we link AD alleles to DNA methylation levels. Finally, we use ChIP-seq to prioritize functional SNPs. As a novel contribution, we present, for the first time, compelling associations between AD-associated risk SNPs and important functional elements at the *APOE* locus (Fig. 1B).

Our investigative journey commenced with a comprehensive exploration of the *APOE* locus, extracting transcriptomic, methylation, and histone modification features from the ROSMAP dorsolateral prefrontal cortex (DLPFC) dataset (see data availability). Serving as our cornerstone, this brain region formed the basis for probing *APOE* gene expression, encompassing bulk tissue RNA-seq (*n* = 573), histone modification through H3K9ac ChIP-seq (*n* = 615), and DNA methylation utilizing the 450 K Illumina array (*n* = 667). Expanding our exploration, we delved into *APOE* locus-associated attributes within two additional brain regions: the posterior cingulate cortex (PCC), comprising a sample size of *n* = 499, and the anterior cingulate cortex (AC), comprising *n* = 433 samples, with the intent of capturing the expression profiles in different brain regions. The overlapped samples across three brain regions can be found in Supplementary Fig. S4. Applying a congruent methodology, the LIBD dataset (see Methods) became another vital resource for investigation. With the DLPFC brain region at its core, this dataset facilitated the accumulation of additional bulk RNA-seq data from European ancestry individuals (*n* = 376) and African Americans (*n* = 216).

Because the vast majority of genes are regulated within an enhancer’s chromosomal position (cis-regulation), we limited our transcriptional mechanism studies to the 2 Mb region [33] containing the *APOE* gene. To select potential functional variants in the selected region, we extracted the genotypes of 6,428 high-quality SNPs from ROSMAP whole-genome sequencing data, 6,483 SNPs from LIBD European, and 10,838 SNPs from LIBD African for downstream analysis.

### *APOE* jxn1.2.2 transcript is uniquely linked to specific AD risk-associated alleles in the *APOE* region

To pinpoint *APOE*'s mRNA transcripts within specific gene regions, we employed an expression feature known as exon-exon junctions. This approach effectively tags specific transcripts, enhancing our ability to quantify them with a heightened degree of precision and specificity, as demonstrated by our recent postmortem brain studies [34–36]. Following the reads alignment and quality controls, our efforts yielded three distinct splicing junctions connecting exon 1 and exon 2, alongside a common junction spanning exon 2 and exon 3, as well as another common junction bridging exon 3 to exon 4 (Fig. 1B). Consequently, our focus homed in on the junction linking alternative exons 1 and 2, a pivotal choice given its capacity to delineate diverse *APOE* transcripts. Then, we combined the *APOE* gene expression information with genomic variants previously selected with the aim to identify the SNPs associated with the levels of the *APOE* transcripts identified. Specifically, we examined the association of selected variants with the global abundance of *APOE* expression (combining reads of all transcripts identified) as well as the abundance of each different spliced isoform. To this end, we conducted a linear regression model implemented in TensorQTL [22]. We used five principal components (PCs) derived from genotype data to correct population stratification, and K PCs derived from expression data to correct potential batch effects (detailed in Methods and Supplementary Table S1 & S2). Across the five RNA-seq datasets (Table 1), we identified an average of 57 k SNP-gene pairs and 5 M SNP-junction pairs at the *APOE* locus, about 6 k and 12 k cis-eQTLs at gene and junction levels with a false discovery rate (FDR) < 0.05.

**Fig. 2:**
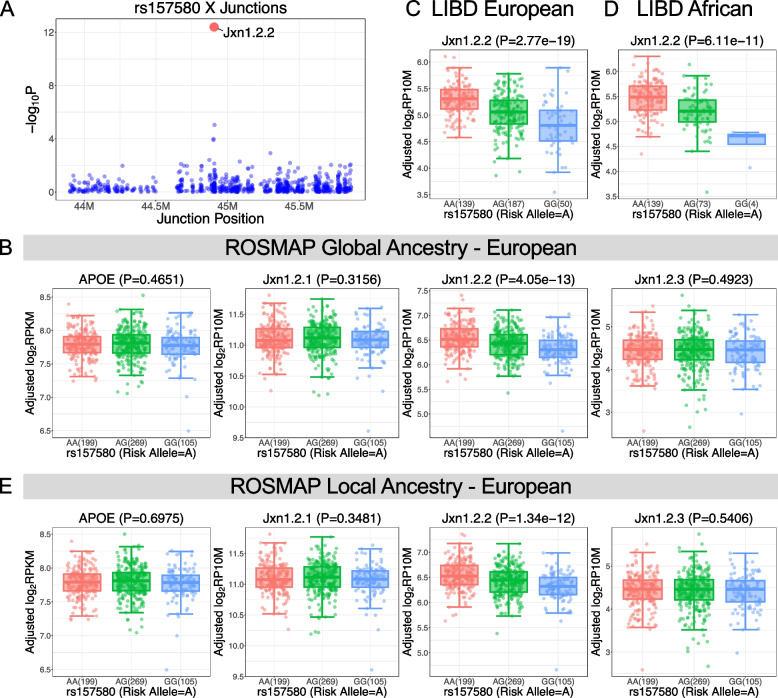
*APOE* jxn1.2.2 transcript is associated with Alzheimer’s disease (AD). **A** jxn1.2.2 expression (red) is the top hit compared to other transcripts at the *APOE* locus (blue) in the ROSMAP brain DLPFC region. The association of AD risk SNP, rs157580, with *APOE* gene level and its 3 transcripts (jxn1.2.1, jxn1.2.2, and jxn1.2.3) in ROSMAP considering global ancestry (**B**). Association of jxn1.2.2 and AD risk SNP in LIBD European ancestry (**C**) and African American (**D**). **E** Local ancestry analysis at the APOE locus

To link the *APOE* transcripts-associated variants (eQTLs) to AD risk alleles, we co-localized observed eQTLs with AD GWAS [5] SNPs. The integration yields an average of 472 SNP-gene pairs and 885 SNP-junction pairs with genome-wide significance for AD risk (*p* < 5e-8) and FDR-significant for eQTL analysis (FDR < 0.05). Importantly, we uncover that a particular junction between alternative exon 1 and exon 2 (named jxn1.2.2 and tagging the *APOE* transcript NM_001302688) is the top hit junction at the *APOE* locus co-localizing with variants associated with AD-risk (*p* < 1e-7) (Figs. 1B and 2A, Supplementary Fig. S5 and Table S5). We didn’t observe statistical significance between AD risk variants (GWAS *p* < 5e-8) and other *APOE* transcripts (jxn1.2.1 and jxn1.2.3) or *APOE* gene-wide expression levels (Fig. 2B, Supplementary Fig. S6A, Table S6 & S7). When we analyzed the other two brain regions, PCC and AC, we found that AD alleles do not influence the jxn1.2.2 transcript expression (Supplementary Table S7). In contrast, the association between the AD alleles and jxn1.2.2 expression was replicated in the LIBD European ancestry brain DLPFC collection (Fig. 2C, Supplementary Fig. S7A & Table S5).

To assess the potential influence of ancestry on the relationship between *APOE* transcripts and AD alleles, we also conducted an analysis of RNA-seq data from the LIBD African ancestry brain DLPFC collections, and this association persists (Fig. 2D, Supplementary Fig. S8A), suggesting a significant link between *APOE* jxn1.2.2 transcripts and AD alleles in samples from two different ancestries. Because we analyzed each European or African population separately to avoid heterogeneity among ancestries, the above analysis was based on global ancestry analysis using Principal Component Analysis (PCA) by integrating genotype data of ROSMAP and LIBD separately with HapMap3 populations (see Methods). Our global ancestry analysis clearly indicated the homogeneous nature of our populations: ROSMAP European, LIBD European and African populations (Supplementary Fig. S9). To further investigate if the results were influenced by population admixture, we performed local ancestry analysis at the *APOE* locus. As expected, the local ancestry results are consistent with our global ancestry analysis (Fig. 2E, Supplementary Figs. S7C & S8C).

The gene structure of *APOE* consists of four exons, with the two SNPs (rs429358 and rs7412 located in exon 4) determining the three common protein isoforms of the *APOE* gene (Fig. 1B). To determine if the association of AD alleles with jxn1.2.2 transcript is independent of the *APOE2,3,4* alleles, we performed the conditional analysis by adding two variables, *APOE4* (4 carriers and non-4 carriers) and *APOE2* (2 carriers and non-2 carriers), in our regression Model-2 (Supplementary Table S2), and found the significant associations were not influenced compared to original model without *APOE4* and *APOE2* in 3 independent datasets: ROSMAP, LIBD European and African populations (Supplementary Figs. S6B & S7B & S8B & S10, Table S6 & S7). Our finding, the association between AD alleles and the jxn1.2.2 transcript is independent of *APOE2,3,4* alleles, was replicated in local ancestry analysis (Supplementary Figs. S7D & S8D). To further define the independent effects of our candidate AD alleles on *APOE* jxn1.2.2 expression from *APOE4* and *APOE2*, we performed epistasis (statistical interaction analysis), and we did not observe significant interactions between our candidate AD alleles and the *APOE4/2* risk allele (Supplementary Fig. S6C & S6D), indicating the association between jxn1.2.2 expression and our candidate AD-risk alleles is not influenced by *APOE4/2*. The independent expression of jxn1.2.2 transcript was further supported by the lack of association between *APOE2,3,4* determining SNPs (rs429358 and rs7412) and jxn1.2.2 expression (Supplementary Table S8).

### *APOE* jxn1.2.2 transcript expression levels are associated with AD pathology, cognitive impairment, and *APOE*4 allele in DLPFC

To explore the role of *APOE* transcripts abundance in AD, we compared its expression level between AD and controls: (1) CERAD criterion to evaluate neuritic plaques [37]. (2) Braak criterion to evaluate the density and distribution of neurofibrillary tangles (NFT) [38, 39]. (3) and in cognitive health [40]. We evaluated mild cognitive impairment (MCI or dcfdx_lv) [41, 42] and cognitive status at the time of death [43] (cogdx). (4) *APOE4* genetic factor [4, 44] by comparing *APOE* gene expression between *APOE4* carriers and *APOE4* non-carriers.

At the gene level by combining all transcripts, the *APOE* expression was marginally significantly associated with cognitive impairment (dcfdx_lv *p* = 0.0166; cogdx, *p* = 0.0432) in DLPFC. However, the *APOE* gene is not differentially expressed in CERAD, braak, and *APOE4* criteria across DLPFC, AC, and PCC brain regions. In addition to neurodegenerative phenotypes, we also compared *APOE* gene expression between neuropsychiatric diseases (schizophrenia, bipolar disorders [BP], major depression disorders [MDD]), and controls in LIBD European and African individuals. However, we didn’t find significant differences (Supplementary Table S9).

**Fig. 3:**
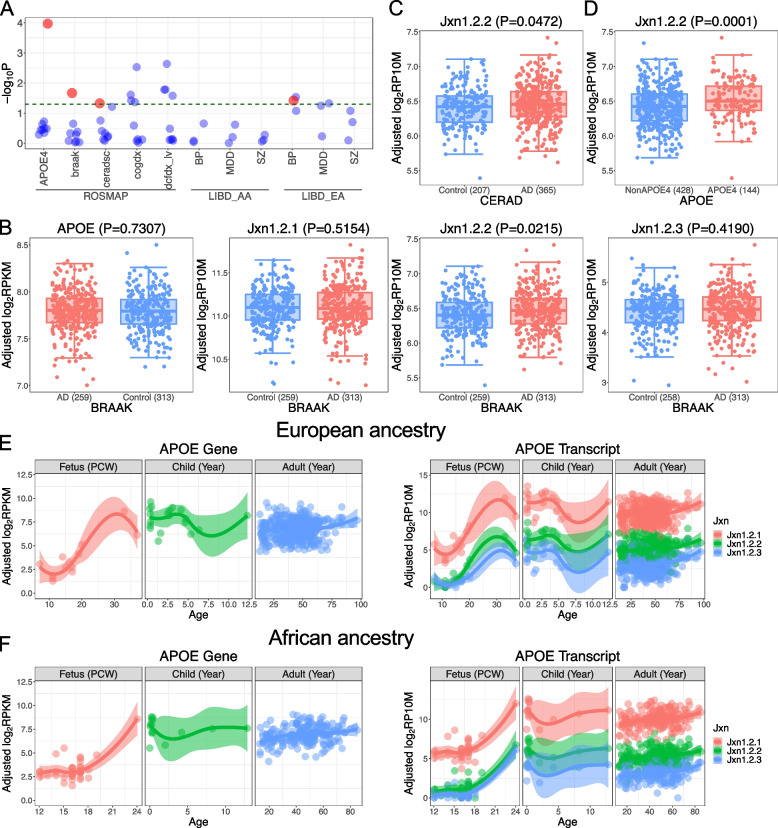
Differential expression of *APOE* at gene level and transcripts level. **A** Differential expression of *APOE* exon-exon junctions across different diagnosis criteria among diverse ethnic groups (European ancestry [EA] and African ancestry [AA]). BP, bipolar disorders; SZ, schizophrenia; MDD, major depression disorders. The dashed line indicates the threshold of *p*-value = 0.05. Bigger red dots are jxn1.2.2. **B** Differential analysis of *APOE* at gene and junction level between AD and controls in BRAAK diagnosis. Differential analysis of jxn1.2.2 transcript in CERAD diagnosis (**C**), *APOE*4 carriers vs. non-carriers (**D**). **E** *APOE* gene and transcripts expression during brain development in brain DLPFC region in European ancestry. **F** *APOE* gene and transcripts expression during brain development in brain DLPFC region in African American ancestry

At the single transcripts level, by analyzing the three transcripts separately, we found that jxn1.2.2 transcript was differentially expressed between AD and controls compared to other *APOE* transcripts in DLPFC (Fig. 3A). *APOE* jxn1.2.2 expression was uniquely associated with amyloid burden as characterized by CERAD pathology (*p* = 0.0472) and NFT characterized by braak pathology (*p* = 0.0215). We did not detect differences for the other *APOE* transcripts (jxn1.2.1 and jxn1.2.3) in DLPFC (Fig. 3B,C). Furthermore, differential jxn1.2.2 expression was observed between *APOE4* carriers compared and non-carriers in European populations from ROSMAP (*p* = 0.0001) (Fig. 3D) and LIBD (*p* = 0.0012, Supplementary Fig. S11A), and the same trend in African population (*p* = 0.0591, Supplementary Fig. S11B). The three transcripts are all significantly associated with cognitive impairment (dcfdx_lv and cogdx *p* < 0.05) (Fig. 3A). In contrast, none of the three transcripts were associated with AD status using the abundance data of PCC and AC brain regions. Additionally, they were not associated with schizophrenia, BP, and MDD in LIBD European and African populations (Supplementary Table S10).

To delineate the expression trajectory of *APOE* transcripts during brain development, we analyzed 227 brain samples across 16 brain regions from 42 human postmortem brains (Supplementary Fig. S2). We plotted the expression patterns of the 3 *APOE* transcripts across 16 brain regions defined by Kang et al. [45] (Supplementary Figs. S12 & S13). We also visualized the expression trajectory by combining all *APOE* transcripts from the 16 brain regions using PC1, which can explain majority of variance (> 67%) (Supplementary Fig. S14). We observed low expression of all the *APOE* transcripts during prenatal stages. They are upregulated during childhood (0 < age < 13). Then, the expression is slightly downregulated during adulthood (13 + years). We replicated the findings of *APOE* expression trajectory in LIBD European postmortem brain DLPFC region (Fig. 3E, Supplementary Table S11). We also found the *APOE* expression trajectory is consistent between European and African ancestries (Fig. 3F). The AD-linked jxn1.2.2 transcript has a medium expression compared to the most abundant transcript jxn1.2.1 and the low-expressed transcript jxn1.2.3 across developmental stages (fetus, child, and adult) across the 16 brain regions (Supplementary Fig. S12 & S13 and Table S4 & S11).

To investigate the differences between the *APOE* transcripts, we aligned the coding sequences of the three transcripts and found distinct 5’ untranslated regions, promoting varied starting points for diverse transcripts. Due to disparate start codon usage, the jxn1.2.2 transcript contained an additional 26 amino acids compared to the other transcripts (Supplementary Fig. S15). To further understand the *APOE* transcripts, we predicted their signal peptides using SignalP 6.0 [46]. While the jxn1.2.1 and jxn1.2.3 isoforms likely possess signal peptides around the 13th amino acid, the jxn1.2.2 retains the same signal peptide following the 26 extra amino acids (Supplementary Fig. S16). Then, we used subprograms, GvH and ALOM, in PSORT2 and predicted the cleavage of the signal peptide in jxn1.2.1 and jxn1.2.3 isoforms at the 19 amino acids, and jxn1.2.2 isoform at the 44 amino acids (Supplementary Table S12). To gain further insight into the *APOE* coding sequences, we performed positive selection analysis, revealing evidence of natural selection upon *APOE* during evolution (see methods in Supplementary file, Figs. S17 & S18, Table S13).

To understand the cell-type-specific regulation of *APOE* levels in the human brain, we analyzed single nucleus RNA-seq data from 46 human postmortem brain DLPFC (European ancestry) focusing on six major cell types **(**see methods in Supplementary file and Fig. S19), we found that *APOE* was significantly upregulated in microglia of AD patients compared to healthy persons in the evaluation of neurofibrillary tangle using braak criterion, amyloid plaque using CERAD criterion, cognitive impairment by MCI and cogdx (Supplementary Fig. S20A, B, C, Table S14). Our results are in line with recent evidence that increased *APOE* expression in microglia has been associated with AD phenotypes [47, 48]. We also observed its differential expression in excitatory neurons when stratified by the *APOE4* allele (Supplementary Fig. S20D), indicating the complex genetic-cellular interactions.

Next, to examine if the jxn1.2.2 transcript encodes a stable protein, we generated a full-length jxn1.2.2-Flag construct that overexpresses the full-length jxn.1.2.2 transcript, with the same transcription initiation site and 5’ UTR found in the endogenous jxn1.2.2 transcript. To assist the detection of protein expression from the jxn1.2.2 transcript, the ORF that potentially encodes a ~ 38 kDa protein was Flag-tagged. Western blot using anti-Flag tag antibodies indicates that the jxn1.2.2 construct, when overexpressed in SK-N-MC cells, is translated into a ~ 38 kDa protein, compared to a positive control encoding a Bb1-Flag protein (Supplementary Fig. S21).

#### Identifying functional SNPs using epigenetic data from brain tissues

**Fig. 4:**
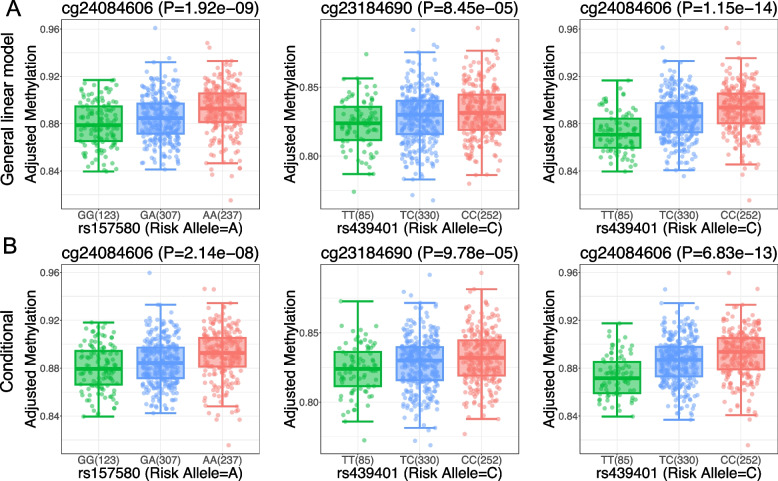
Genotypic impact of candidate SNPs on DNA methylation levels in ROSMAP DLPFC. **A **Association of the candidate AD risk SNPs with CpG sites. **B **The association of the AD-linked CpGs is not affected by the *APOE* 2&4 allele by conditional analysis

To identify potential regulatory SNPs in the *APOE* region, we carried out a rigorous statistical effort to identify CpGs spanning the *APOE* region. We obtained 788 CpG sites and performed association analysis between 7,937 SNPs and methylation levels in selected epigenetic features (mQTL). After filtering with mQTL FDR < 0.05, we obtained 4,640 SNPs and 221 CpG sites. Subsequently, to link the DNA methylation with AD, we integrated selected CpG sites with AD variants and eQTL results. We identified 17 CpG significantly associated with 31 SNPs that reached GWAS significance (*p* < 5e-8) and are associated with jxn1.2.2 abundance (FDR < 0.05) (Supplementary Table S5). We observed significant impacts of AD alleles on CpG methylation (FDR < 0.05) (Fig. 4A). To determine whether the effect of DNA methylation can be modified by the *APOE4* and *APOE2* alleles, we performed conditional analysis by including the *APOE4* and *APOE2* as co-variants, and found the results were not influenced (Fig. 4B). We also checked for statistical interaction between methylation levels and AD alleles. As expected, we did not observe significant interactions between our candidate AD alleles and *APOE4* and *APOE2* on the DNA methylation levels (Supplementary Fig. S22). Consistent with the independent relationship, we found that *APOE2,3,4* determining SNPs are not associated with our prioritized CpG methylation levels (Supplementary Table S8).

**Fig. 5:**
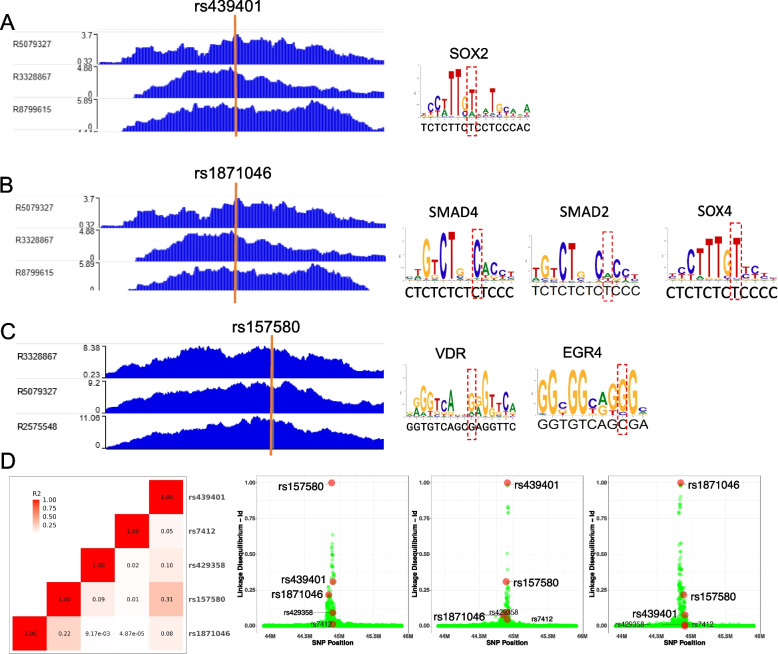
Candidate SNPs at the *APOE* locus located within active chromatin affect transcriptional factors (TFs)’ binding affinity. Left panel: (**A**) rs439401, (**B**) rs1871046, and (**C**) rs157580 are co-localized with H3K9ac ChIP-seq peak from human postmortem brains. Right panel: Recognition sites of TFs involved in Alzheimer’s disease are influenced by the 3 SNPs. The red dash box indicates the binding site of each SNP. **D** Linkage disequilibrium of candidate SNPs with other SNPs spanning *APOE*, including the two *APOE*2,3,4-determining SNPs

ChIP-seq experiments can determine which chromatin regions are actively involved in gene transcription. From the above analysis, we have identified 31 SNPs associated with jxn1.2.2 transcript expression and DNA methylation (meQTL). Here we carried out several steps to prioritize SNPs within active chromatin at the *APOE* locus: First, we identified 7 SNPs located within active chromatin regions by co-localizing the H3K9ac ChIP-seq peaks with the 41 SNPs. Second, most enhancers exert their regulatory function through the binding of TFs. Thus, we performed an in-silico search of the DNA sequence of the 7 SNPs for putative TF binding sites using Motif Scan and Enrichment Analysis (MoSEA) and removed 1 SNP with no motif binding. Third, we reviewed the literature and found motifs affected by 3 SNPs (rs1871046, rs157580, and rs439401) that were reported to be involved in neuronal function (Fig. 5A, B, C, Supplementary Table S15). We predicted that SOX4 and SMAD TF family members would bind to rs1871046. SOX2 would bind to rs439401. rs157580 was predicted to be located within binding sites of EGR4 and vitamin D receptor (VDR).

The 3 candidate SNPs were not significantly associated with global *APOE* levels in European and African populations across our 5 datasets (Supplementary Table S6). However, they were associated with the jxn1.2.2 transcript (FDR < 0.05) (Supplementary Table S7). Among the 3 SNPs associated with jxn1.2.2 expression levels in European cohorts, two SNPs (rs157580 and rs439401) were also significantly associated with jxn1.2.2 expression levels in African, indicating the shared regulatory mechanisms for both ancestries. To check the relationship between the 3 SNPs, we performed linkage disequilibrium and found they are relatively independent (weak correlation) (Fig. 5D). For example, *r*^2^ of the meQTLs with rs439401 in European is less than 0.4 (Supplementary Table S5). Importantly, the 3 SNPs may represent partially independent meQTLs associated with AD risk, according to the weak linkage disequilibrium with the common AD-risk polymorphisms (rs7412 and rs429358 defining the *APOE2,3,4* alleles, Fig. 5D). CSF Amyloid-beta 42 (Aβ42) and phosphorylated tau (pTau) are two major proteins implicated in the AD pathological process that can be assayed. We studied the genetic effects on CSF Aβ42 and pTau levels in a total of 13,116 individuals using GWAS data [49]. We found that rs157580 and rs439401 SNPs are associated with both biomarkers in CSF (*p* = 4.37e-74 and 1.97e-58 separately), while rs1871046 is weakly associated (*p* = 1.64e-3) (Supplementary Fig. S23). Our epistasis analysis confirmed that *APOE2&4* have no significant effects on the correlation between the two SNPs (rs157580 and rs439401) and DNA methylation (*p* > 0.05) (Supplementary Fig. S22). Summary-based Mendelian Randomization (SMR) can evaluate the mediation effect of gene expression on association between SNP and phenotype [31]. To further demonstrate the causal effects of the alleles on the expression of *APOE* transcripts, we performed SMR and the results are consistent (*p* < 1e-7) (Supplementary Table S16). To expand our observation to other neurological diseases, we investigate the 3 SNPs we prioritized and the two *APOE*2,3,4 determining SNPs across GWAS of neurodegenerative (e.g., Parkinson’s disease) and neuropsychiatric disorders (e.g., schizophrenia). Interestingly, we found those SNPs are specifically associated with AD (Supplementary Fig. S24 and Table S17).

## Discussion

The *APOE2&4* alleles are the strongest but not the only genetic risk factors for AD. Indeed, GWAS[5] has identified numerous potential AD-risk SNPs. However, the molecular mechanism of most AD loci remains largely elusive. Despite *APOE* has long been a widely investigated gene since the identification of its association with lipid levels and AD, the biological mechanisms behind these associations are unknown. Many studies have reported the relationship between APOE2,3,4 protein isoforms and AD-related traits, such as impairing synaptic repair and plasticity [50], increasing beta-amyloid aggregation [51–53], increasing formation of neurofibrillary tangles, and decreasing metabolic activity of neurons [54]. These phenotypes have been largely attributed to APOE2,3,4 protein isoform biochemical properties that differ by single amino acid substitutions constituted by alleles of rs7412 and rs429358 [55]. Indeed, beyond the overt differential molecular bending of APOE2,3,4 isoforms and subsequent alterations in lipidation capacity [56, 57], there is limited evidence supporting functional variants at this locus modulating full-length *APOE* isoforms.

Here, we provide evidence of additional functional elements at the *APOE* locus that may contribute to the mechanism of action of the *APOE* locus in AD and related phenotypes. We leveraged data from multiple large population-based cohorts of human postmortem brains in diverse ethnic groups. Our study offers insights into the genomics elements controlling *APOE* expression in the brain, but the pathological relevance of observed *APOE* transcripts by including/excluding exons and their regulatory mechanism will need additional clarifications in the future. Similar to our work in *SNX19* [35] and *CYP2D6* [34] genes, we demonstrate that a careful analysis of postmortem brain data can identify brain region-specific gene transcription mechanisms associated with AD-risk *APOE*. Our results prioritize specific domains between exon 1 and exon 2 in the protein that contain the functional domain that might influence AD risk. The data made us aware that the AD susceptibility signals can also be masked in gene expression analysis, and that the focus on individual transcripts is absolutely crucial to understanding *APOE* mechanisms operating not only in the brain but also in other tissues expressing this pleiotropic gene. Furthermore, pinpointing additional functional mechanisms modulating causal common variants at the *APOE* region and elucidating their roles in AD susceptibility might contribute to delineating therapeutic strategies for controlling this important susceptibility factor. Unfortunately, controlling *APOE*-associated risk remains a major challenge of dementia research. Our results, therefore, refine our understanding of the *APOE* locus and suggest that multiple variants affecting *APOE* regulatory motifs might have independent effects influencing AD susceptibility. Although the AD-linked *APOE* jxn1.2.2 mRNA is associated by *APOE4* genotype, the association between AD-risk SNPs (e.g., rs157580) we prioritized and jxn1.2.2 expression is independent of *APOE4* genotype, indicating an alternative mechanism of *APOE* mRNA transcription. We have illustrated the relationship between *APOE* jxn1.2.2 mRNA, rs157580, and *APOE4* in Supplementary Fig. 25.

Strengths of this study include the use of the ROSMAP cohort in our main analyses, replicated and extended in the LIBD cohort and its connection with large meta-GWAS of AD risk. The ROSMAP brain collections are unique in terms of the ages of the subjects involved, and the donors have been followed longitudinally [17]. This study is also strengthened by identifying the potential pathogenic role of *APOE* jxn1.2.2 transcript, and replicating it in the two additional cohorts with different ancestries. Importantly, this transcript is also a risk expression feature in the African ancestry population. Despite we characterize *APOE* transcripts in three regions (DLPFC, PCC, and AC) of the brain, the *APOE* jxn1.2.2 transcript was differentially expressed between AD and controls and in the DLPFC brain region. The DLPFC is a region affected by amyloid-β pathology relatively early as it spreads throughout the neocortex [58]. The accumulation of tau pathology progresses stereotypically captured by the braak stages [59], and the DLPFC displays an accumulation of neurofibrillary tangles containing tau typically when individuals begin to be symptomatic. Thus, both pathological amyloid-β and tau accumulate in the DLPFC in AD, and we use quantitative measures of these pathologies to enhance our power in discovering the molecular features that are associated with these pathologies. However, we feel that characterizing more brain regions and studying the expression of this specific transcript in other human organs in multiple independent cohorts are necessary to understand its potential role in AD pathogenesis and its connection with mature APOE protein isoforms.

A major finding of this study is that the *APOE* jxn1.2.2 transcript might differentially contribute to AD risk compared to other alternative transcripts. We found that AD alleles are specifically associated with enhanced jxn1.2.2 expression. Consistently, we also found that upregulation of *APOE* jxn1.2.2 transcript is associated with AD hallmarks (amyloid burden and NTF). Those findings support our hypothesis that AD-linked *APOE* transcript signals can be masked in analysis at the gene level. Given that cognitive deficit was associated with both *APOE* gene and transcript levels, we suggest all the *APOE* transcripts share effects on the cognitive phenotype. Hence, it might be an important additional factor within this region. To the best of our knowledge, this is the first study to pinpoint this AD-linked *APOE* coding mRNA transcript. We propose that this transcript may be regulated by AD SNPs in a disease-state manner or could itself be driven by AD pathology. Because the current association studies were exclusively performed in adult populations (age > 13), our findings can only be representative of this group. *APOE* expression trajectories across brain development stages indicate that the jxn1.2.2 transcript may not be activated (low expression) during early brain development stages. Larger fetus postmortem brain studies are helpful to establish the correlation between *APOE* expression and AD risk alleles in the stages of prenatal development.

Although we analyzed the *APOE* transcripts using brain RNA-seq data, whether transcripts can be detected largely depends on the expression levels. For instance, in our recent postmortem brain study, we discovered that the transcript skipping exon 9 is linked to disease risk. However, our deep long-read sequencing identified multiple transcripts having an exon 9 skip [35]. Long-read deep sequencing would facilitate a comprehensive understanding of the full spectrum of the *APOE* transcripts in human brain tissue. We have pinpointed that the jxn1.2.2 transcript has an extra peptide at the 5’ gene region, initiated by a distinct start codon. Future investigations focusing on this peptide may yield novel insights into the mechanisms by which *APOE* contributes to AD. Considering that our research and others have suggested *APOE*'s potential involvement in AD through microglia, researchers could overexpress the three transcripts in microglia to explore the role of diverse *APOE* transcripts in molecular and cellular phenotypes (e.g., a-beta accumulation). Although we evaluated *APOE* at the gene level with neuropathological hallmarks, a major caveat of our study is that, due to the relative abundances from 3′ biased 10 × data, it is still challenging to evaluate the causative effects at the transcript level.

Despite the wealth of evidence linking *APOE* SNPs to pathology implicated in AD, an understanding of the specific mechanism(s) by which genetic variation at this region alters risk remains incomplete. *APOE* acts in conjunction with other genetic and environmental factors to confer AD risk. DNA methylation and chromatin status are associated with genetic and environmental factors, and previous studies have identified associations with AD and neuropathological hallmarks of AD in large collections of human brain tissue samples [60, 61]. However, DNA methylation at the *APOE* locus has not been well studied. We found *APOE* alleles associated with AD and associated simultaneously with methylation levels of 3 CpG sites that were also reported to be involved in other brain phenotypes: cg24084606, located in *APOC1P1* promoter, was also reported to be associated with *TOMM40* expression in postmortem brain hippocampus [62]. There was a weak association between cg24084606 and autism spectrum disorder in a South African Cohort [63]. A Scottish Family Health Study showed that cg23184690 is differentially methylated between *APOE4* carriers and *APOE2* carriers [64]. The evidence suggests that epigenetic elements dynamically and spatially influence diverse gene expression, contingent upon genetic background, disease status, and tissue types.

Chromatin accessibility has been shown to play a crucial role in AD and other neurological diseases. H3K9ac marks transcriptionally active open chromatin and has been shown to be associated with AD in human postmortem brains [65]. This experiment indicates that our prioritized genetic variants may actively be involved in transcriptional regulation. Interestingly, our data suggests that members from *SOX* and *SMAD* families might play a role during *APOE* gene transcription. These TFs were previously reported to be involved in AD dementia [66], tau pathology-mediated cognitive dysfunction [67], and β-amyloid levels in the brain [68]. However, the binding of one TF alone is rarely enough to directly infer functional effects on the gene expression levels, typically under the combinatorial and dynamic control of multiple TFs. Therefore, TF data are often actively integrated with other functional genomic techniques to decipher the basic regulatory control of gene expression, such as by incorporating active chromatin regions, DNA methylations, and SNPs. Interestingly, the 2 AD GWAS SNPs we prioritized are located within active open chromatin regions, correlated with CpG methylation levels, influence *APOE* jxn1.2.2 transcript expression, and have genetic effects on AD core features in CSF (Aβ42 and pTau). Indeed, Bekris et al. demonstrated that *APOE* promoter activity is significantly influenced by an active chromatin region, *TOMM40* IVS2-4 [69], in which region rs157580 is located (Supplementary Fig. S26). It would be interesting to investigate the allele effects of the 3 SNPs in human induced pluripotent stem cells (hiPSCs)-derived neurons, microglia, and astrocytes.

Our study revealed new *APOE* gene regulatory mechanisms affecting common AD risk SNPs that may interact with chromatin, TFs, and DNA methylation to be responsible for turning the *APOE* transcription on or off in a different set of cells, or at different times. Though we identified several potential functional variants associated with AD risk in this study, we still do not know how this genetic control of gene expression confers AD risk and pathology. It is likely that these identified SNPs affect the *APOE* jxn1.2.2 expression level no matter the *APOE* genotype, and the change of *APOE* jxn1.2.2 expression may play a pivotal role in neuropathogenesis. Finally, this work also highlights the importance of including different ancestries in research on AD, as shared functional elements can provide windows of opportunity to cure the disease in diverse populations.

## Conclusion

We utilized multi-omics data from five independent human postmortem brain datasets, encompassing both European and African populations across three brain regions. Our findings reveal that the expression of the *APOE* jxn1.2.2 specific transcript is a shared feature among these two ethnic groups. By analyzing epigenomic data, we identified several AD-risk SNPs that are linked with DNA methylation, chromatin status, and transcription factor binding. Given that both the transcript and the genomic elements controlling its expression are novel, we suggest that they may serve as additional targets for AD therapeutics and preventive measures aimed at mitigating the AD risk associated with *APOE*.

### Supplementary Information

Supplementary Figures: Figure S1. GWAS summary statistics at the *APOE* locus (hg38, chr19:44,655,791−45,159,393). Color is coded for linkage disequilibrium of predicted functional SNPs (rs1871046, rs157580, and rs439401) and SNPs consisting of APOE2,3,4 genotypes (rs429358 and rs7412). There are 33 SNPs (including rs429358) with *p*-value = 0. In order to plot in locuszoom, we labeled these SNPs with *p*-value = 1e-300. Supplementary Figure S2. BrainSpan Samples for the study of *APOE* expression trajectory during brain development. The full name of the brain region is in Supplementary Table S4. Supplementary Figure. S3. Data processing and relationship. WGS, whole genome sequencing. Supplementary Figure S4. Overlapped Samples in ROSMAP. (A) Overlapped Samples with RNA-Seq Data in Different Brain Regions. (B) Overlapped Samples with RNA-Seq Data and DNA Methylation Data in ROSMAP DLPFC brain region. Brain region: DLPFC, dorsolateral prefrontal cortex; PCC, posterior cingulate cortex; AC, anterior cingulate cortex. Supplementary Figure S5. rs439401 and rs157580 are associated with *APOE* jxn1.2.2 transcript compared to other expression features at this locus in DLPFC brain region using global ancestry analysis. Supplementary Figure S6. Genotypic impact of candidate SNP, rs439401, on *APOE* expression at gene and transcripts levels in ROSMAP DLPFC considering global ancestry. (A) Association of rs439401 with expression of *APOE* features. (B) Conditional analysis of rs439401 by considering *APOE4* and *APOE2*. *APOE4* (C) and *APOE2* (D) alleles have no interaction with rs439401 on *APOE* expression. Supplementary Figure S7. Genotypic impact of candidate SNP, rs439401, on *APOE* expression at gene and transcripts levels in LIBD DLPFC from European Ancestry. (A) Association of rs439401 with *APOE* expression features considering global ancestry. (B) Conditional analysis of rs439401 by considering *APOE4* and *APOE2* alleles considering global ancestry. (C) Association of rs439401 with *APOE* expression features considering local ancestry. (D) Conditional analysis of rs439401 by considering *APOE4* and *APOE2* alleles in local ancestry analysis. Supplementary Figure S8. Genotypic impact of candidate SNP, rs439401, on *APOE* expression at gene and transcripts levels in LIBD DLPFC from African Americans. Association of rs439401 with *APOE* expression features considering global ancestry (A) and local ancestry (C). Conditional analysis of rs439401 by considering *APOE*2,3,4 alleles and global ancestry (B) and local ancestry (D). Supplementary Figure S9. Principal component analysis (PCA) for (A) HapMap populations (reference), (B) ROSMAP European ancestry, (C) LIBD European ancestry, and (D) African American. The ancestry score shows our populations are homogenous after removing outliers. Supplementary Figure S10. Conditional analysis of *APOE* expression features with rs157580 considering global ancestry. EA, European ancestry; AA, African American. Supplementary Figure S11. Differential expression of *APOE* at gene level and transcripts level between *APOE4* carriers and non-carriers in LIBD (A) European and (B) African American. Supplementary Figure S12. *APOE* transcripts expression during brain development in 16 brain regions. Note: check the full name of each brain region in Supplementary Table S4. Supplementary Figure S13. Averaged expression of the 3 *APOE* transcripts in each brain region across different developmental stages. The full names of brain regions are in Supplementary Table S4. Supplementary Figure S14. *APOE* transcripts expression during brain development represented by PC1 summarizing 16 brain regions. PCW, post-conceptional weeks. Supplementary Figure S15. Diverse 5’ untranslated region and coding sequences of *APOE* transcripts. Supplementary Figure S16. Predicted signal peptide of *APOE* transcripts. Supplementary Figure S17. Alignment of *APOE* coding sequences across species. Supplementary Figure S18. *APOE* evolutionary tree across species. Supplementary Figure S19. Single nucleus sequencing of human brains recapitulates cell-type-specific marker genes (A) and cell types (B). Supplementary Figure S20. Differential expression of *APOE* across 6 cell types in DLPFC between AD and healthy controls in (A) braak stages, (B) CERAD criterion, (C) cognitive impairment, and (D) between *APOE4* carriers vs. non-carriers. Supplementary Figure S21. The *APOE* jxn1.2.2 transcript is translatable in SK-N-MC cells. (A) Anti-Flag western Blot detected the expression of a Flag-tagged protein in SK-N-MC cells transfected with the APOE jxn1.2.2-Flag construct, as quantified in (B). A Bb1-Flag construct was used as a positive control. The Flag-tagged protein/b-actin ratio in SK-N-MC cells transfected with Bb1-Flag was normalized to 1. Mean ± SEM are shown. One-way ANOVA and Tukey post hoc test, ****p<0.0001. Supplementary Figure S22. Epistasis analysis detects interaction between (A) *APOE4*, (B) *APOE2*, and candidate SNP on DNA methylation levels. Supplementary Figure S23. A-beta protein GWAS summary statistics at the *APOE* locus (hg38, chr19:44,655,791−45,159,393). Color is coded for linkage disequilibrium of predicted functional SNPs (rs1871046, rs157580, and rs439401) and SNPs consisting of *APOE*2,3,4 genotypes (rs429358 and rs7412). Supplementary Figure S24. *APOE* functional SNPs across GWAS of neurodegenerative and neuropsychiatric disorders. AD, Alzheimer’s disease; PD, Parkinson’s disease; ALS, Amyotrophic Lateral Sclerosis; ADHD, Attention-deficit/hyperactivity disorder; ASD, Autism Spectrum Disorders; ANX, anxiety disorder; BIP, bipolar disorders; MDD, major depression disorders; PTSD, post-traumatic stress disorder; SCZ, schizophrenia. See GWAS data source in Supplementary File. Supplementary Figure 25. The relationship between rs157580, jxn1.2.2 mRNA, *APOE4*, and *APOE*2,3,4-determining SNPs (rs429358 & rs7412). Supplementary Figure S26. *APOE* locus LD plot. *APOE* locus linkage disequilibrium (LD) plot shows the strong LD between *TOMM40* and *APOE*. Higher numbers in squares represent stronger LD calculated using D’. Highlighted regions represent strong haplotype blocks. rs157580 is in the TOMM40 intervening sequence (IVS) 2-4 enhancer region.  Plot was generated using ROSMAP European ancestry genotype data with Haploview. Haplotype regions for promotors and enhancers inserted into luciferase reporter constructs were labeled according to *Bekris et al*. (PMID: 22089642).

Supplementary Tables: Table S1. Summary statistics for each RNA-Seq dataset. Supplementary Table S2. Analysis models. Supplementary Table S3. *APOE* genotypes are derived from two *APOE* SNPs. Supplementary Table S4. *APOE* expression trajectory during brain development in BrainSpan data. Supplementary Table S5. Summary statistics of GWAS, eQTL, mQTL across DLPFC, PCC, and AC brain regions at the *APOE* locus. Supplementary Table S6. Association of candidate SNPs with *APOE* gene level expression. Supplementary Table S7. Association of candidate SNPs with abundance of specific *APOE* gene transcripts. Supplementary Table S8. Association of *APOE*2,3,4 determining SNPs (rs429358 and rs7412) with features including *APOE* gene expression, *APOE* transcripts expression, and DNA methylation. Supplementary Table S9. Differential analysis of *APOE* gene between Alzheimer’s disease and controls across ROSMAP DLPFC, AC, and PCC brain regions; between schizophrenia/bipolar disorders/major depression disorders (MDD) and controls in LIBD Caucasian and African Americans. Supplementary Table S10. Differential analysis of junction between APOE exon 1 and exon 2 between Alzheimer's disease and controls across ROSMAP DLPFC, AC, and PCC brain regions; between schizophrenia/bipolar disorders/major depression disorders (MDD) and controls in LIBD Caucasian and African Americans. Supplementary Table S11. *APOE* expression trajectory during brain development in LIBD data. Supplementary Table S12. Signal peptide prediction of *APOE* transcripts. Supplementary Table S13. *APOE* positive selection LRT and positive selection sites. Supplementary Table S14. Differential expression of*APOE* gene in six major brain cell types across diverse diagnoses. Supplementary Table S15. Transcriptional factors binding to our candidate SNPs. Supplementary Table S16. Summary-based Mendelian randomization (SMR) of rs157580 and rs439401 with *APOE* jxn1.2.2 transcript. Supplementary Table S17. GWAS summary statistics of candidate SNPs.

Supplementary Methods and Results.

## Data Availability

WGS, bulk and single nucleus RNA-seq, DNA methylation, and ChIP-seq data from ROSMAP are available via the AD Knowledge Portal (Project SynID: syn2580853; https://adknowledgeportal.org), subject to requirements for data access and data attribution. GWAS summary data of AD dementia is available from https://fundacioace-my.sharepoint.com/:u:/g/personal/iderojas_fundacioace_org/EaTwlPg9cRJHn7Kos4h39OUBaxajsjJHL_C110fC89bc8w?e=ZdcEUy. See other GWAS Summary Statistics Source in Supplementary File.

## Abbreviations

AD: Alzheimer’s disease
GWAS: Genome-wide association studies
SNPs: Single nucleotide polymorphisms
DLPFC: Dorsolateral prefrontal cortex
PCC: The posterior cingulate cortex
AC: The anterior cingulate cortex
ROSMAP: The Religious Orders Study/Memory and Aging Project
LIBD: The Lieber Institute for Brain Development
LD: Linkage disequilibrium
eQTLs: Expression quantitative trait loci
PC: Principal components
FDR: False discovery rate
IBD: Identity by Decent test
PCA: Principal Component Analysis
NFT: Neurofibrillary tangles
MCI: Mild cognitive impairment
cogdx: Cognitive status at the time of death
BP: Bipolar disorders
MDD: Major depression disorders
MoSEA: Motif Scan and Enrichment Analysis
WGS: Whole-genome sequencing
MHC: Major Histocompatibility Complex
