# Supplementary material for: Identification of a specific APOE transcript and functional elements associated with Alzheimer’s disease
Source: Mol Neurodegener. 2024 Aug 29;19:63. doi: 10.1186/s13024-024-00751-7 (PMC11361112; doi:10.1186/s13024-024-00751-7)
Supplement: Supplementary file 1 — Supplementary Figures: Figure S1. GWAS summary statistics at the APOE locus (hg38, chr19:44,655,791−45,159,393). Color is coded for linkage disequilibrium of predicted functional SNPs (rs1871046, rs157580, and rs439401) and SNPs consisting of APOE2,3,4 genotypes (rs429358 and rs7412). There are 33 SNPs (including rs429358) with p-value = 0. In order to plot in locuszoom, we labeled these SNPs with p-value = 1e-300. Supplementary Figure S2. BrainSpan Samples for the study of APOE expression trajectory during brain development. The full name of the brain region is in Supplementary Table S4. Supplementary Figure. S3. Data processing and relationship. WGS, whole genome sequencing. Supplementary Figure S4. Overlapped Samples in ROSMAP. (A) Overlapped Samples with RNA-Seq Data in Different Brain Regions. (B) Overlapped Samples with RNA-Seq Data and DNA Methylation Data in ROSMAP DLPFC brain region. Brain region: DLPFC, dorsolateral prefrontal cortex; PCC, posterior cingulate cortex; AC, anterior cingulate cortex. Supplementary Figure S5. rs439401 and rs157580 are associated with APOE jxn1.2.2 transcript compared to other expression features at this locus in DLPFC brain region using global ancestry analysis. Supplementary Figure S6. Genotypic impact of candidate SNP, rs439401, on APOE expression at gene and transcripts levels in ROSMAP DLPFC considering global ancestry. (A) Association of rs439401 with expression of APOE features. (B) Conditional analysis of rs439401 by considering APOE4 and APOE2. APOE4 (C) and APOE2 (D) alleles have no interaction with rs439401 on APOE expression. Supplementary Figure S7. Genotypic impact of candidate SNP, rs439401, on APOE expression at gene and transcripts levels in LIBD DLPFC from European Ancestry. (A) Association of rs439401 with APOE expression features considering global ancestry. (B) Conditional analysis of rs439401 by considering APOE4 and APOE2 alleles considering global ancestry. (C) Association of rs439401 with APOE expressio [file 13024_2024_751_MOESM1_ESM.pdf]

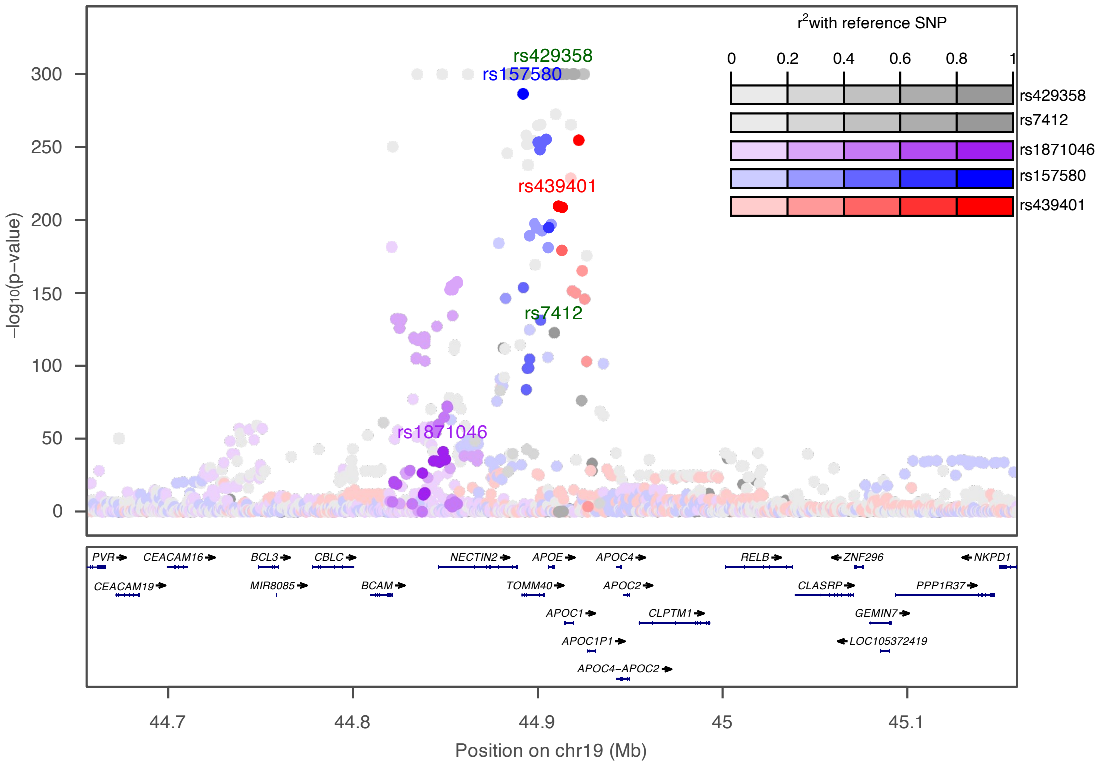

Supplementary Figure S1. GWAS summary statistics at the *APOE* locus (hg38, chr19:44,655,791−45,159,393). Color is coded for linkage disequilibrium of predicted functional SNPs (rs1871046, rs157580, and rs439401) and SNPs consisting of APOE2,3,4 genotypes (rs429358 and rs7412). There are 33 SNPs (including rs429358) with p-value = 0. In order to plot in locuszoom, we labeled these SNPs with p-value = 1e-300.

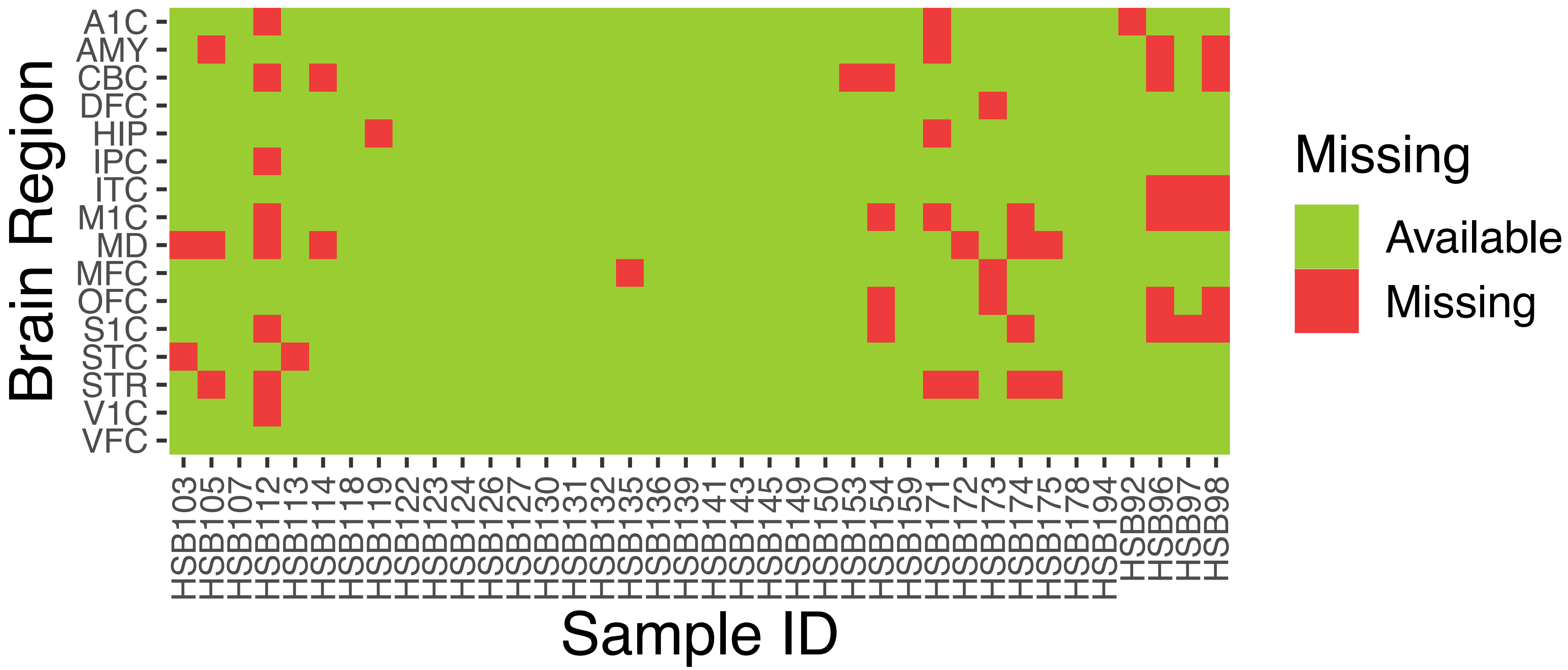

Supplementary Figure S2. BrainSpan Samples for the study of *APOE* expression trajectory during brain development. The full name of the brain region is in Supplementary Table S4.

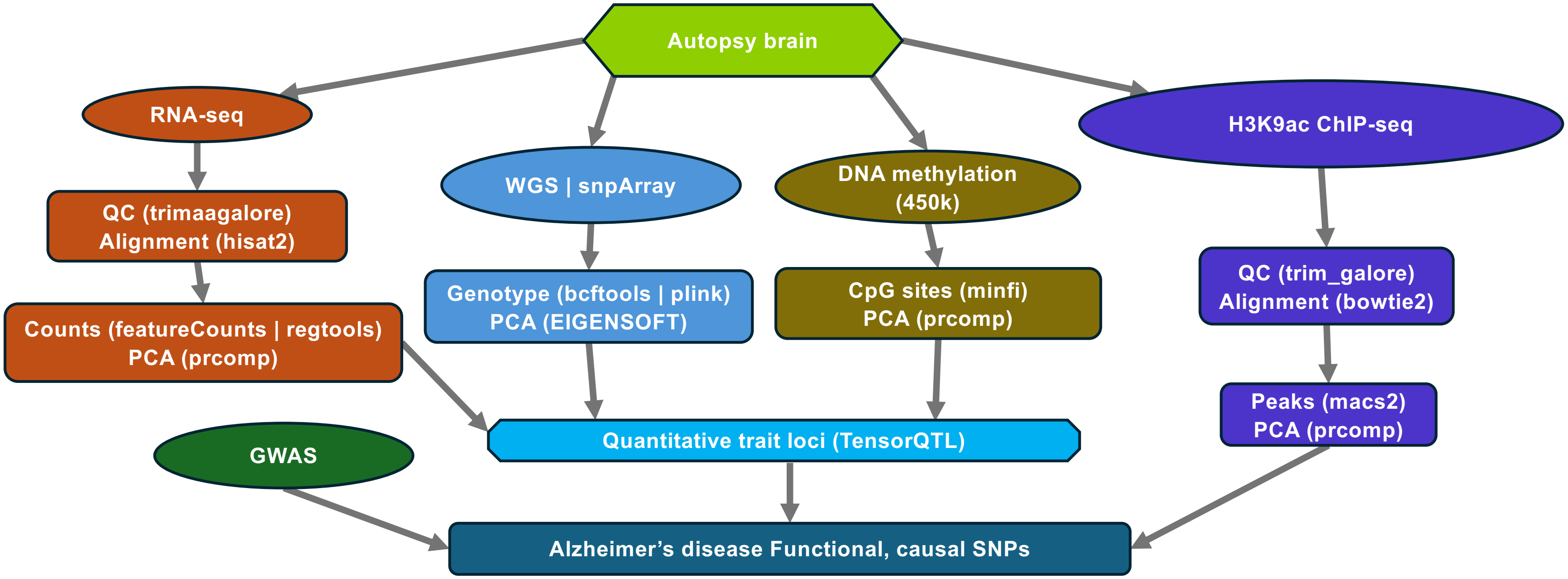

Supplementary Figure. S3. Data processing and relationship. WGS, whole genome sequencing

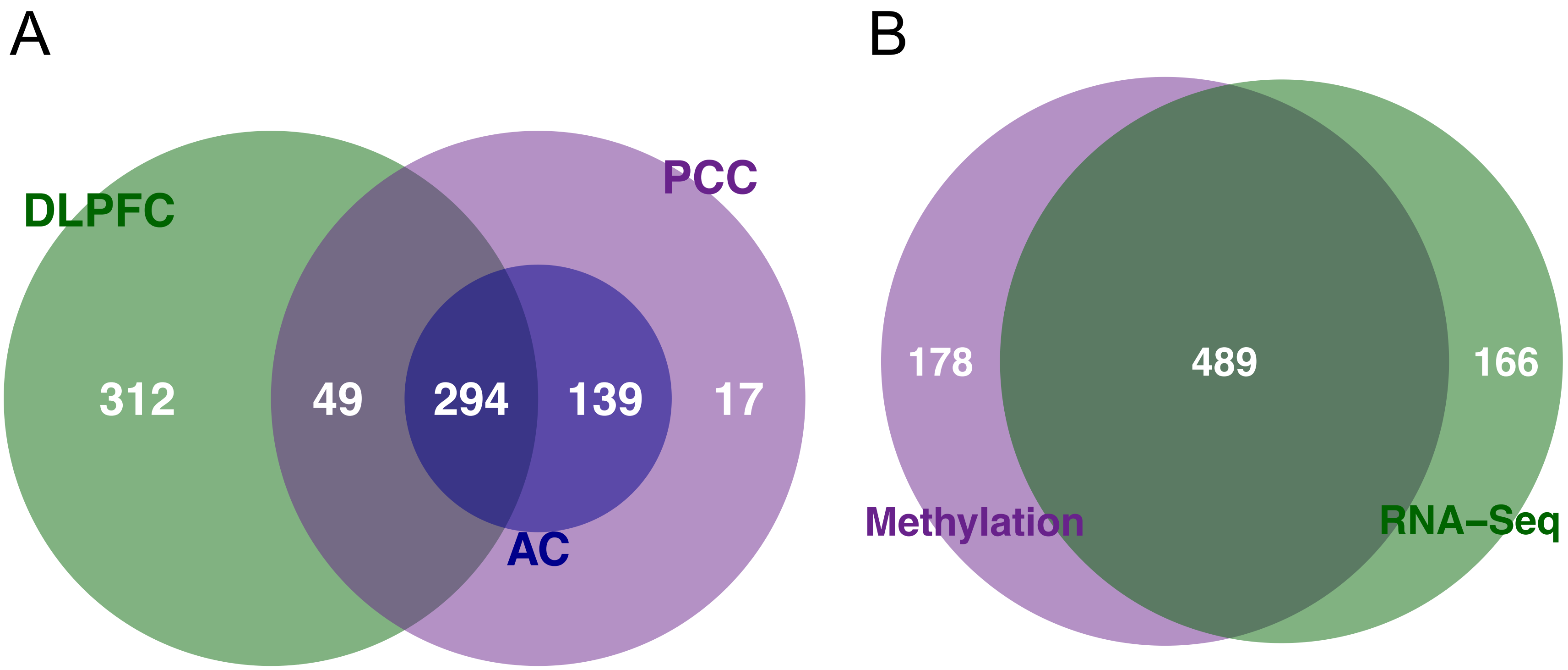

Supplementary Figure S4. Overlapped Samples in ROSMAP. (A) Overlapped Samples with RNA-Seq Data in Different Brain Regions. (B) Overlapped Samples with RNA-Seq Data and DNA Methylation Data in ROSMAP DLPFC brain region. Brain region: DLPFC, dorsolateral prefrontal cortex; PCC, posterior cingulate cortex; AC, anterior cingulate cortex.

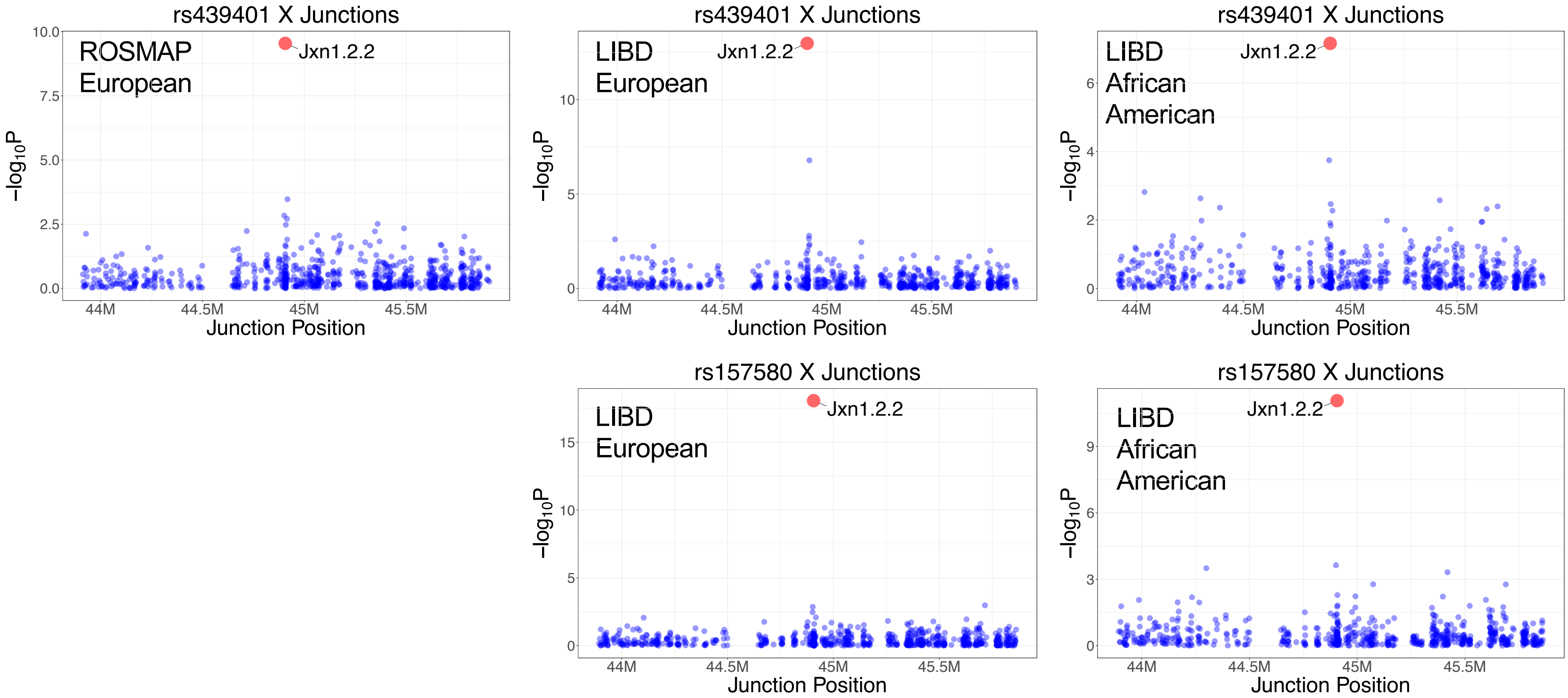

Supplementary Figure S5. rs439401 and rs157580 are associated with *APOE* jxn1.2.2 transcript compared to other expression features at this locus in DLPFC brain region using global ancestry analysis.

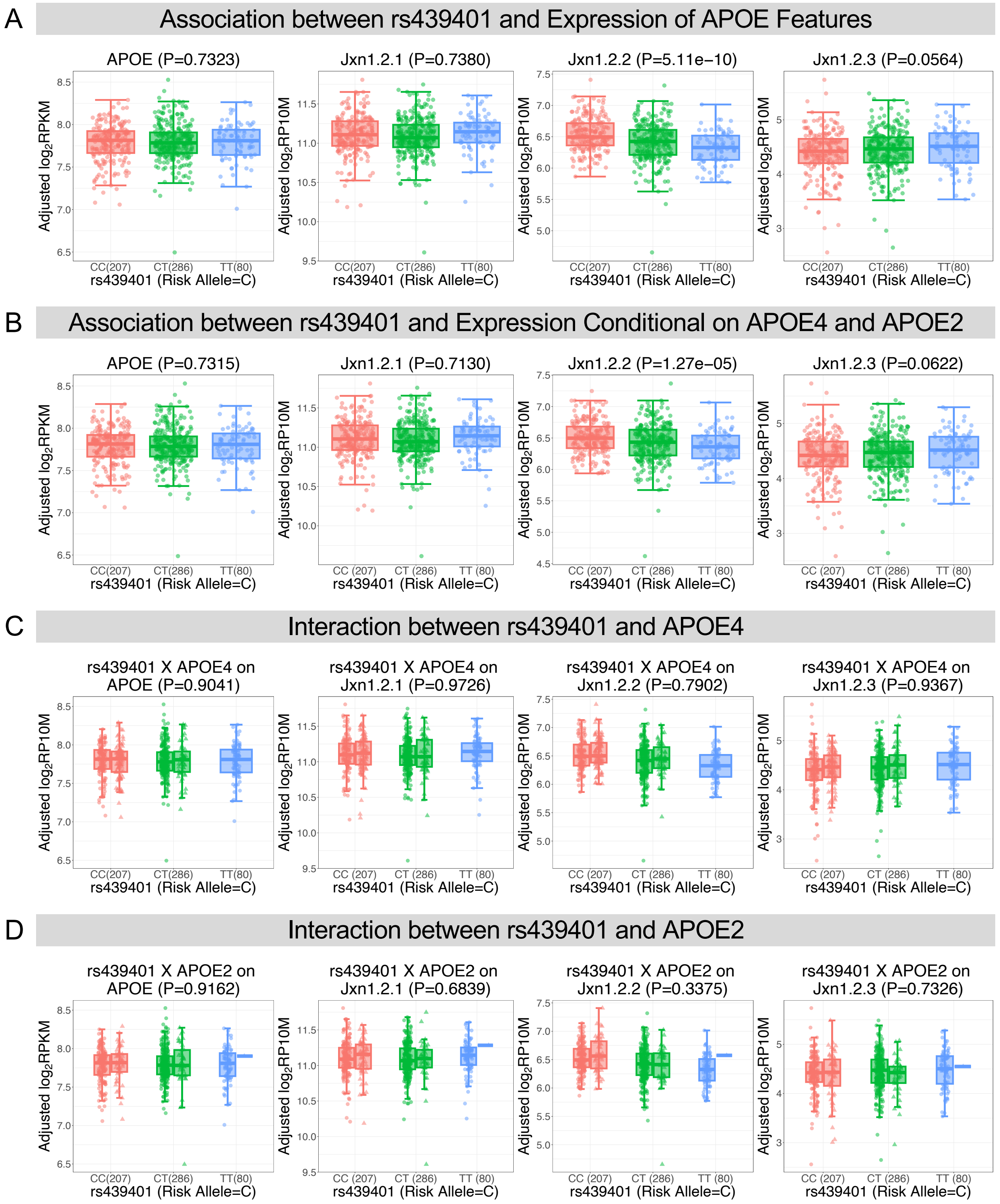

Supplementary Figure S6. Genotypic impact of candidate SNP, rs439401, on *APOE* expression at gene and transcripts levels in ROSMAP DLPFC considering global ancestry. (A) Association of rs439401 with expression of *APOE* features. (B) Conditional analysis of rs439401 by considering *APOE4* and *APOE2*. *APOE4* (C) and *APOE2* (D) alleles have no interaction with rs439401 on *APOE* expression.

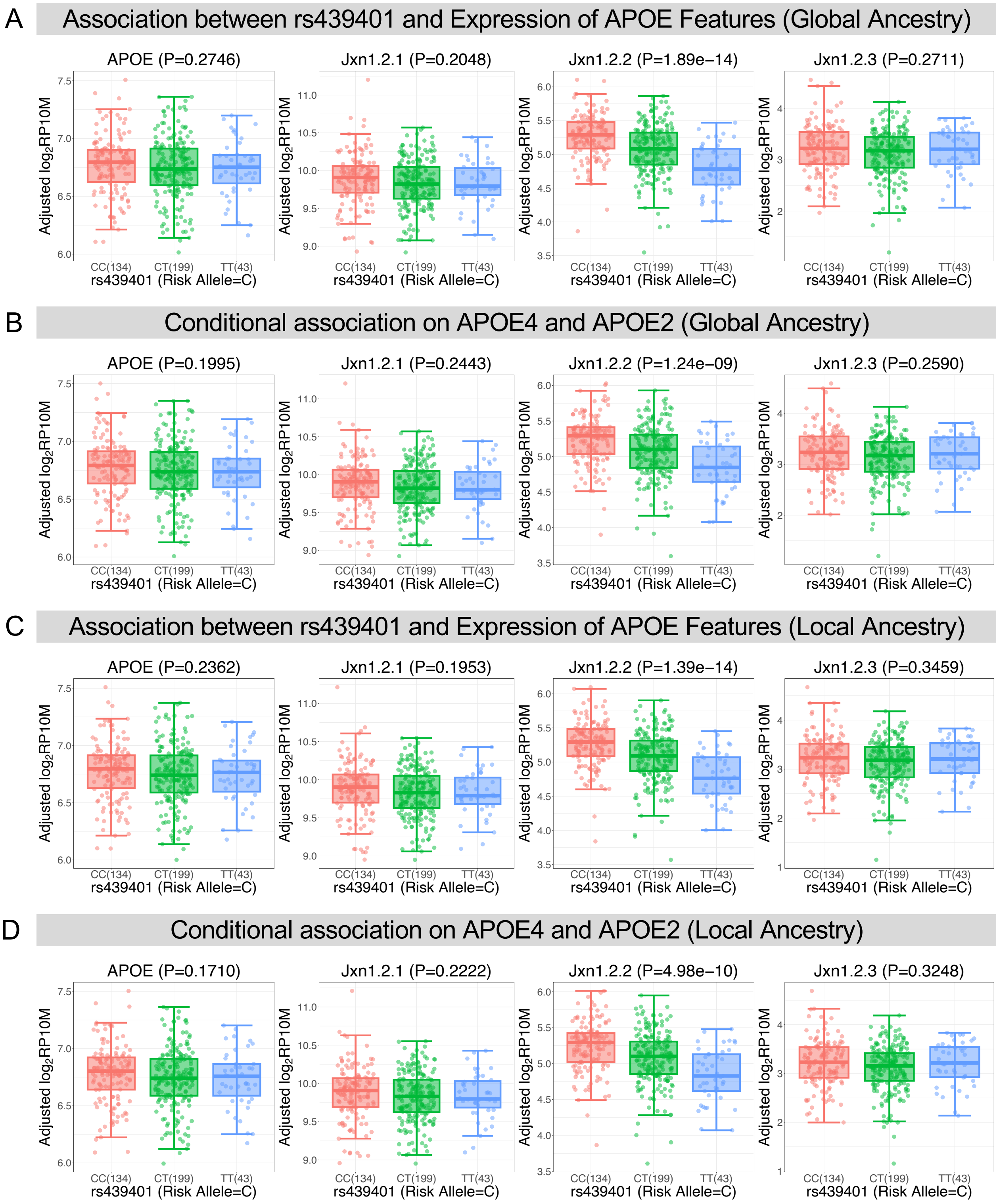

Supplementary Figure S7. Genotypic impact of candidate SNP, rs439401, on *APOE* expression at gene and transcripts levels in LIBD DLPFC from European Ancestry. (A) Association of rs439401 with *APOE* expression features considering global ancestry. (B) Conditional analysis of rs439401 by considering *APOE4* and *APOE2* alleles considering global ancestry. (C) Association of rs439401 with *APOE* expression features considering local ancestry. (D) Conditional analysis of rs439401 by considering *APOE4* and *APOE2* alleles in local ancestry analysis.

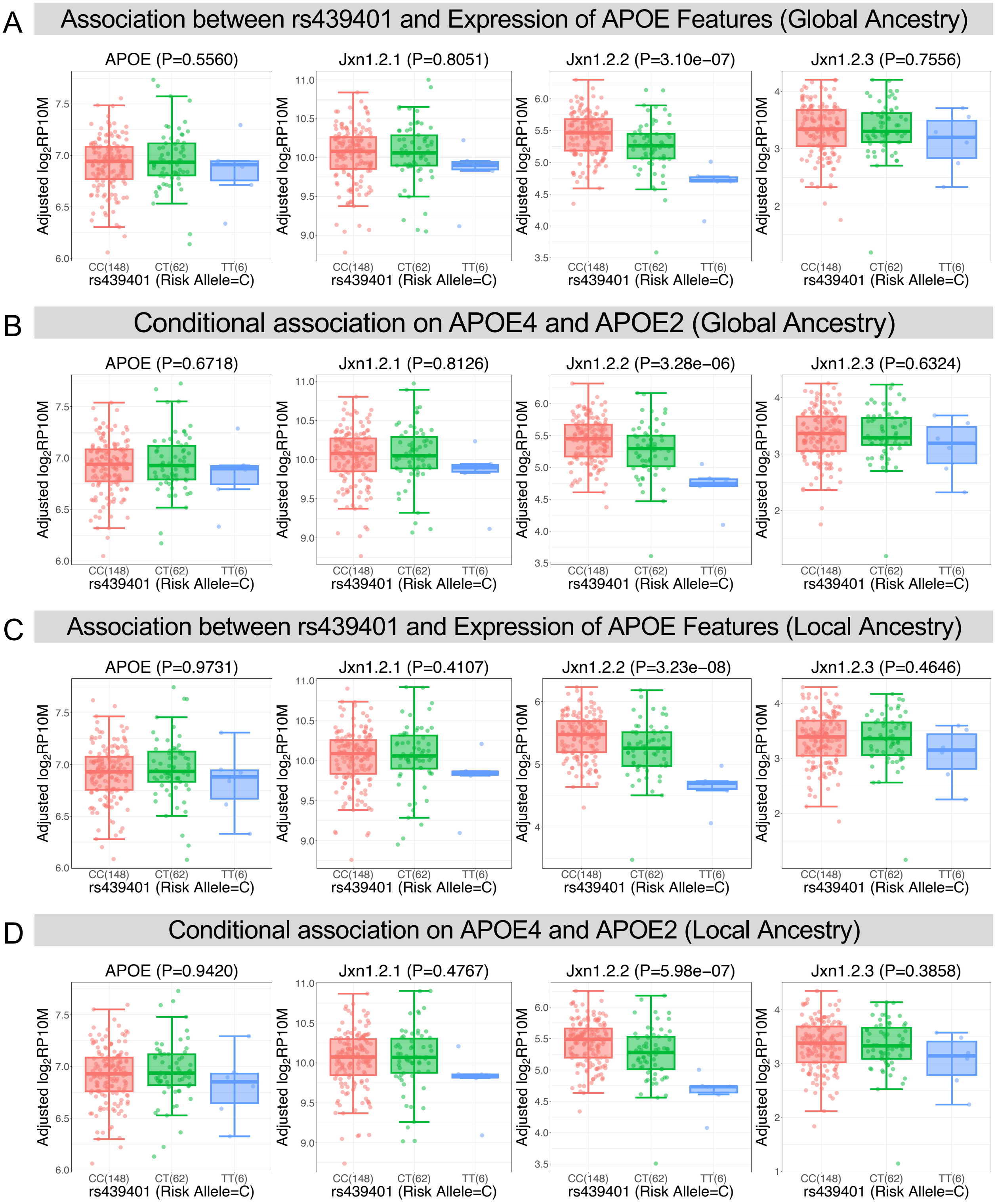

Supplementary Figure S8. Genotypic impact of candidate SNP, rs439401, on *APOE* expression at gene and transcripts levels in LIBD DLPFC from African Americans. Association of rs439401 with *APOE* expression features considering global ancestry (A) and local ancestry (C). Conditional analysis of rs439401 by considering *APOE*2,3,4 alleles and global ancestry (B) and local ancestry (D).

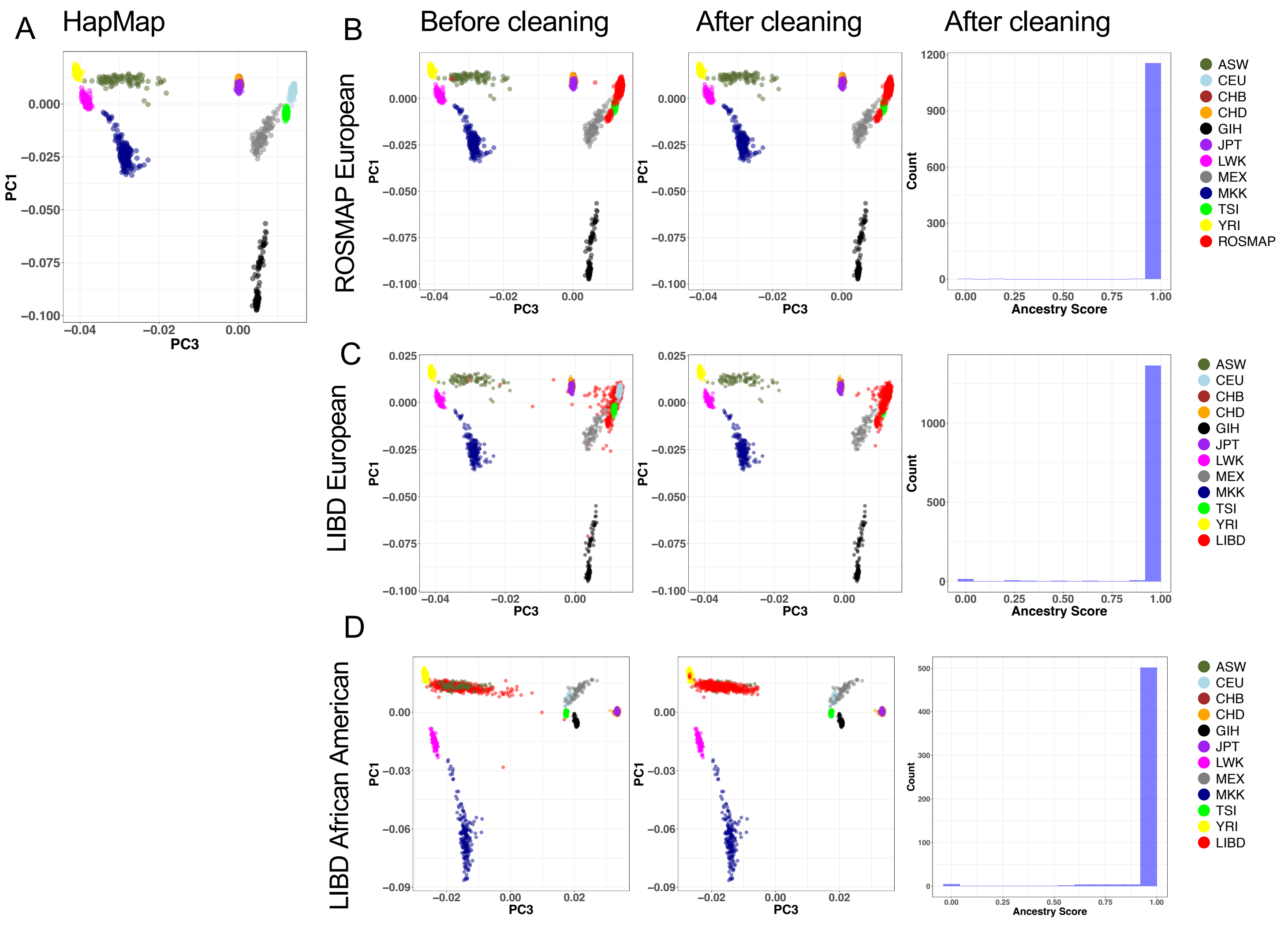

Supplementary Figure S9. Principal component analysis (PCA) for (A) HapMap populations (reference), (B) ROSMAP European ancestry, (C) LIBD European ancestry, and (D) African American. The ancestry score shows our populations are homogenous after removing outliers.

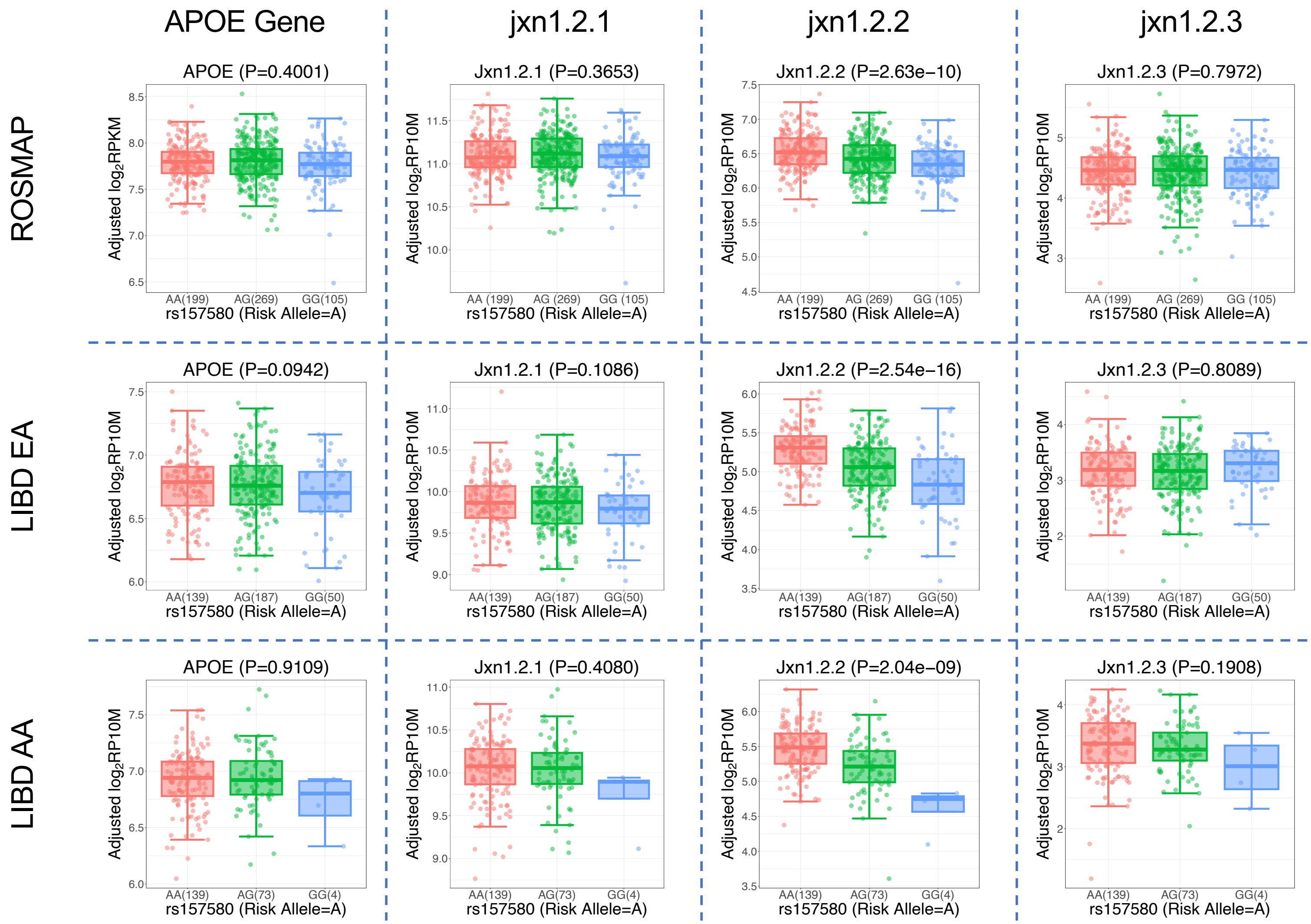

Supplementary Figure S10. Conditional analysis of *APOE* expression features with rs157580 considering global ancestry. EA, European ancestry; AA, African American

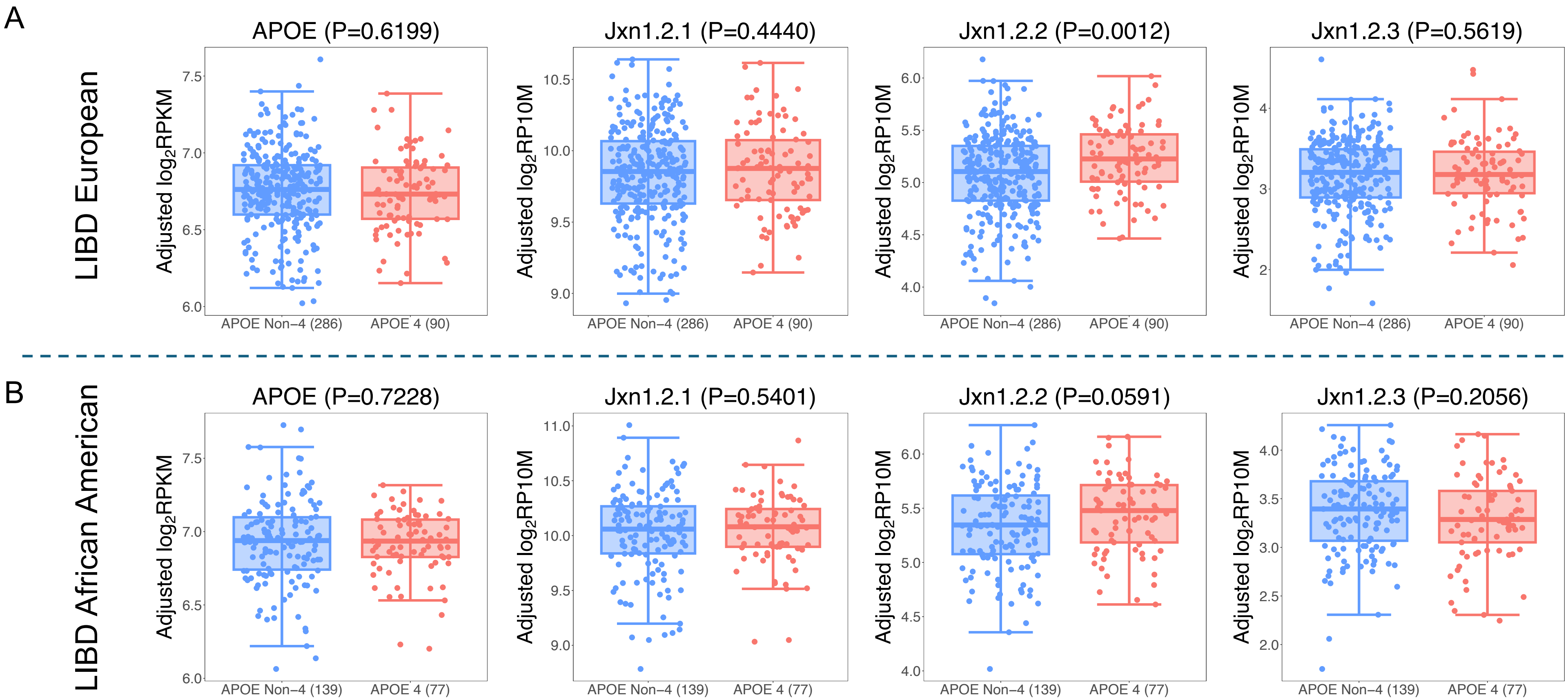

Supplementary Figure S11. Differential expression of *APOE* at gene level and transcripts level between *APOE4* carriers and non-carriers in LIBD (A) European and (B) African American.

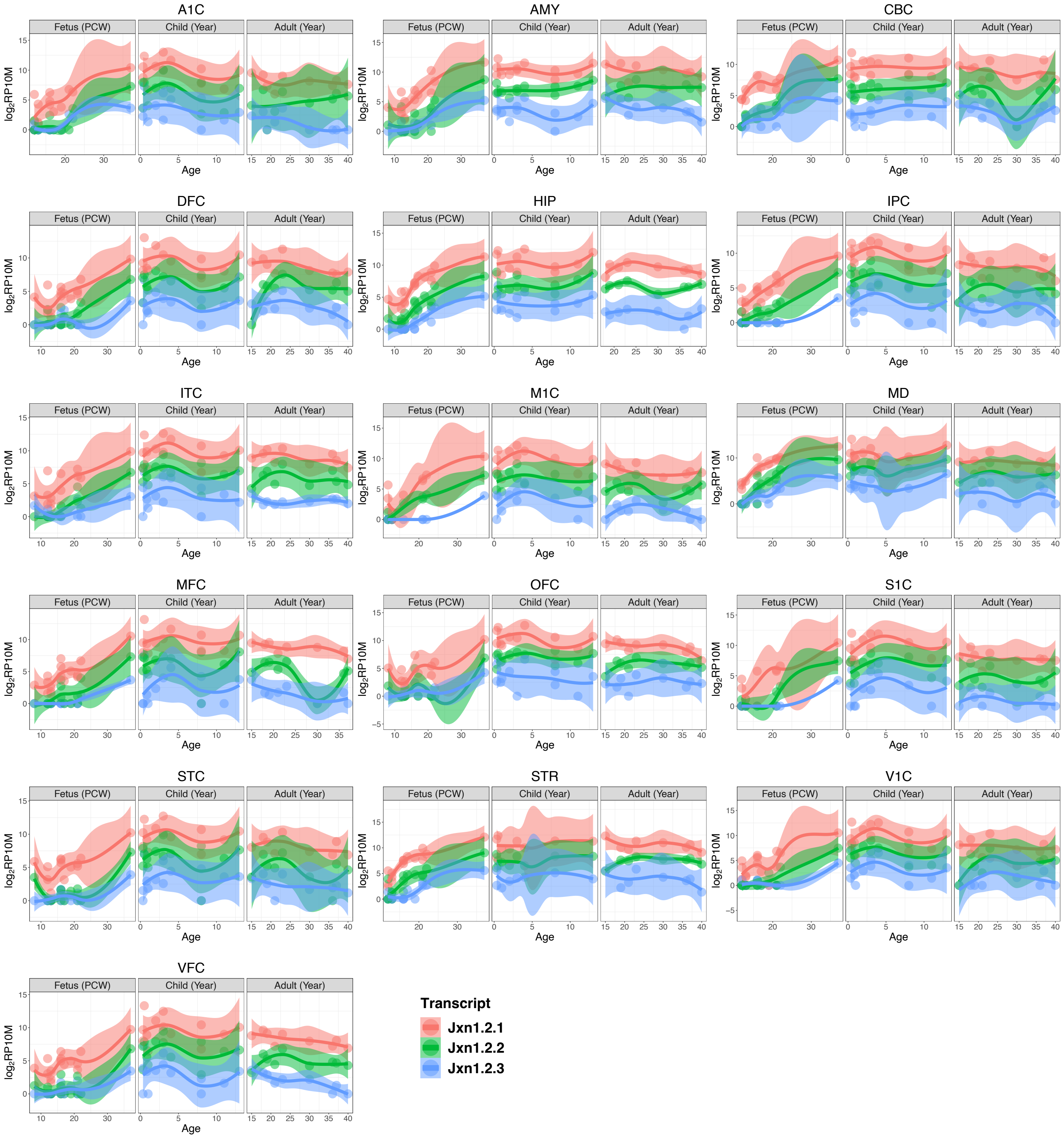

Supplementary Figure S12. *APOE* transcripts expression during brain development in 16 brain regions. Note: check the full name of each brain region in Supplementary Table S4.

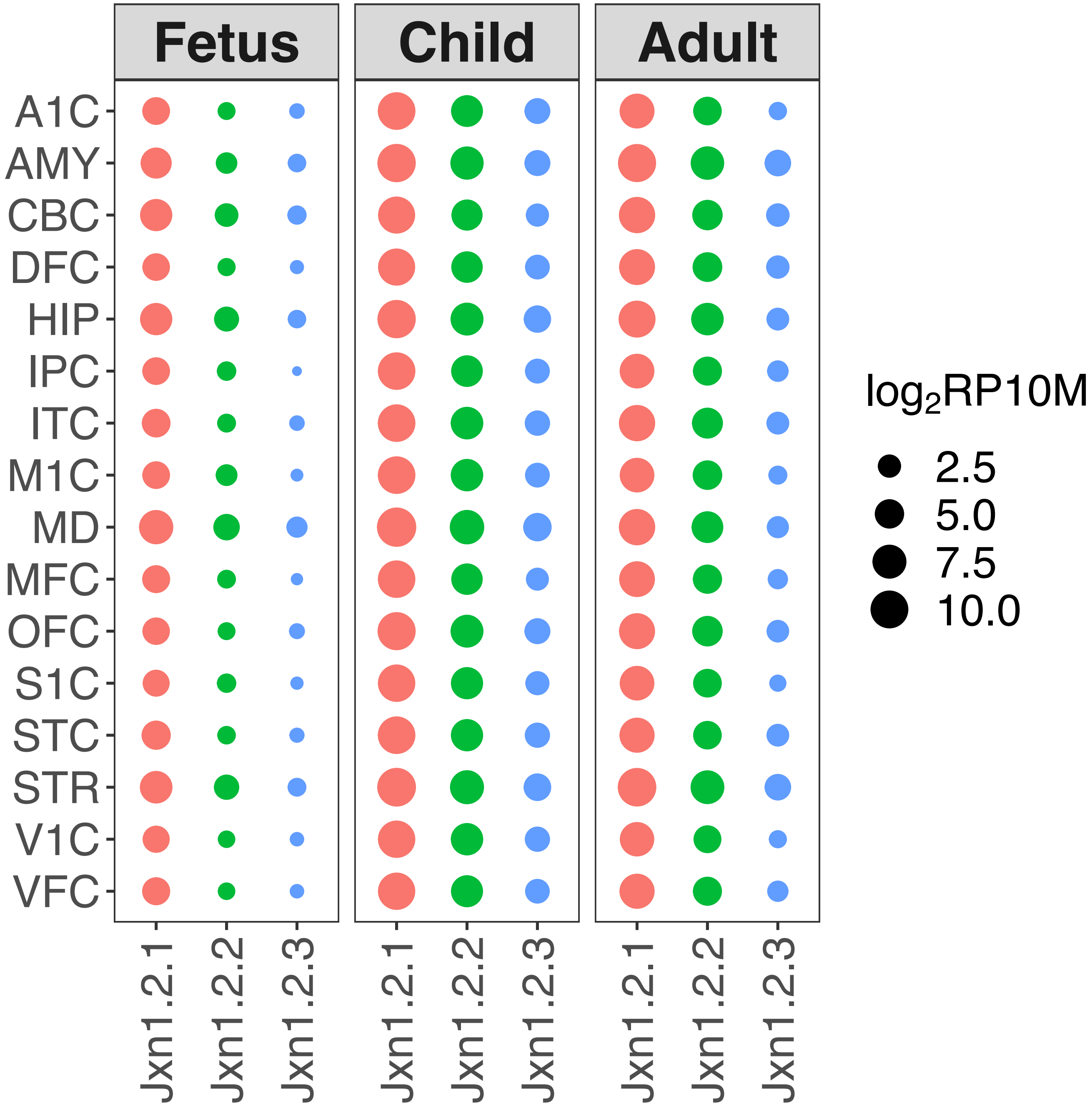

Supplementary Figure S13. Averaged expression of the 3 *APOE* transcripts in each brain region across different developmental stages. The full names of brain regions are in Supplementary Table S4.

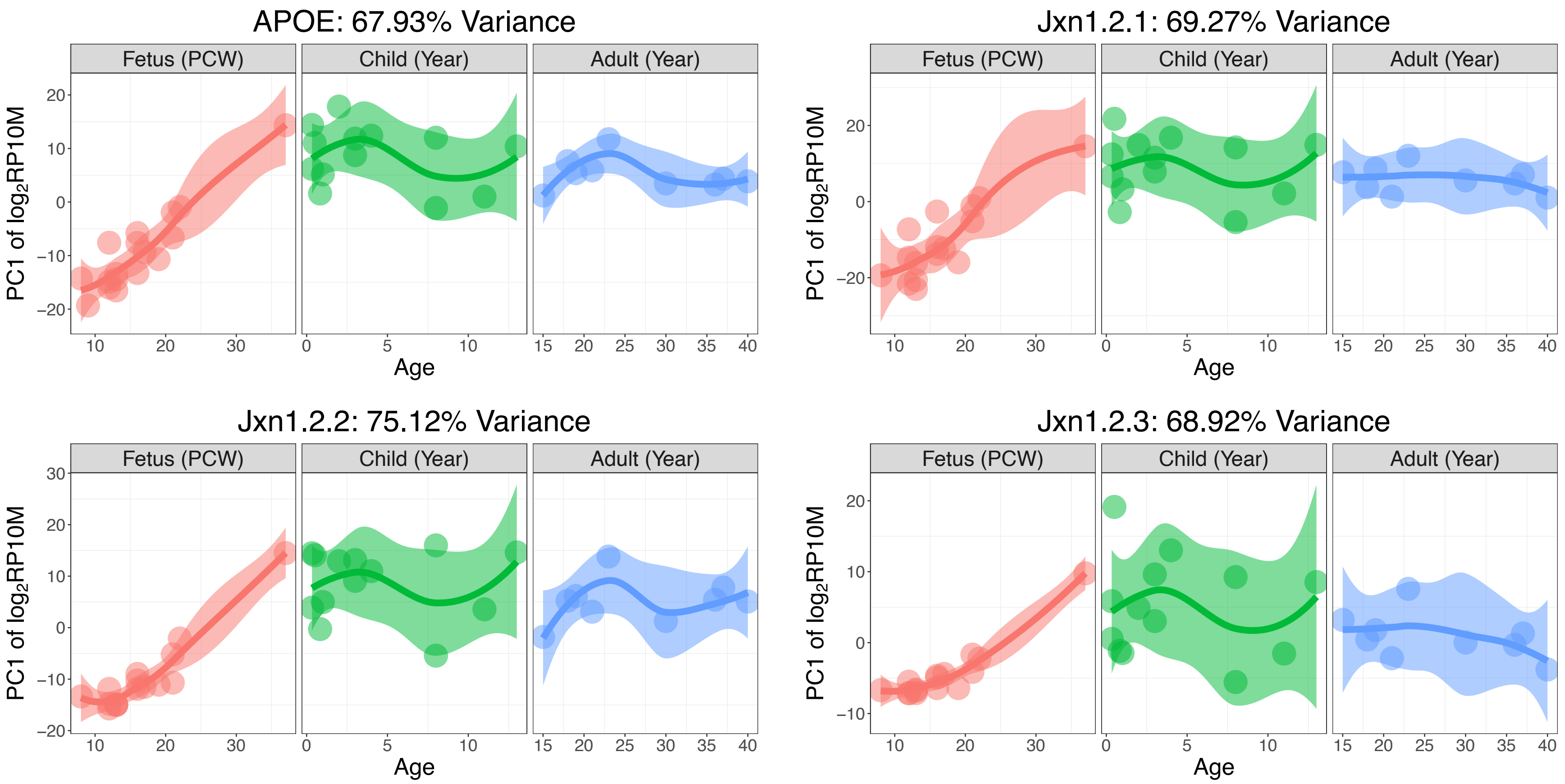

Supplementary Figure S14. *APOE* transcripts expression during brain development represented by PC1 summarizing 16 brain regions. PCW, post-conceptional weeks.

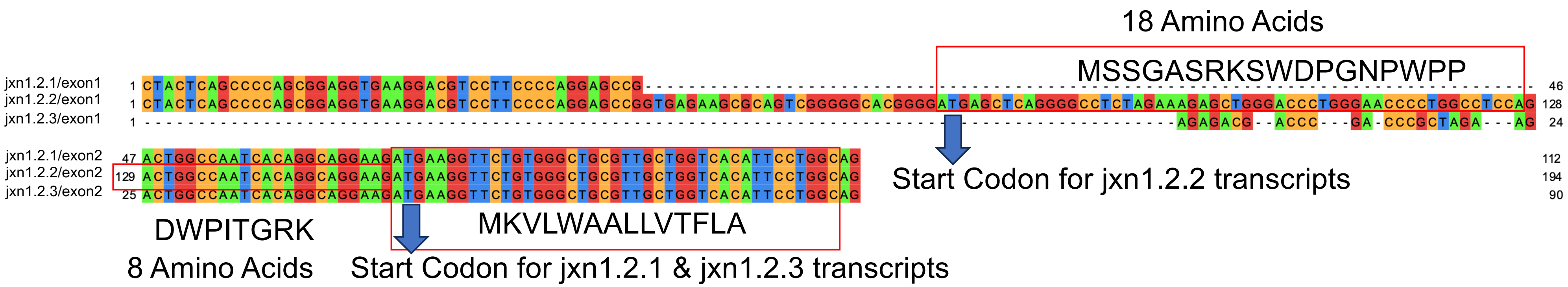

Supplementary Figure S15. Diverse 5’ untranslated region and coding sequences of *APOE* transcripts.

jxn1.2.1
jxn1.2.3

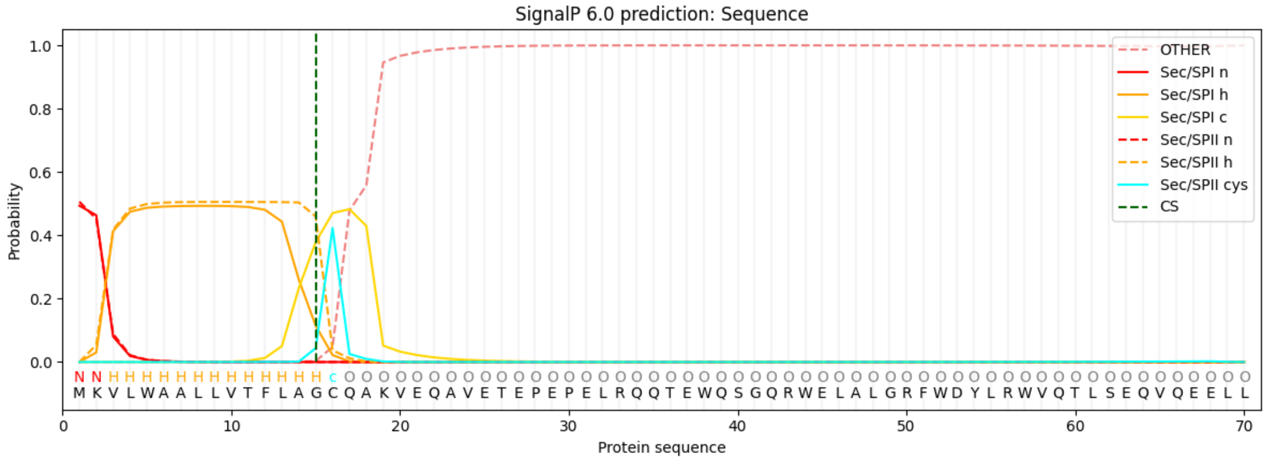

jxn1.2.2

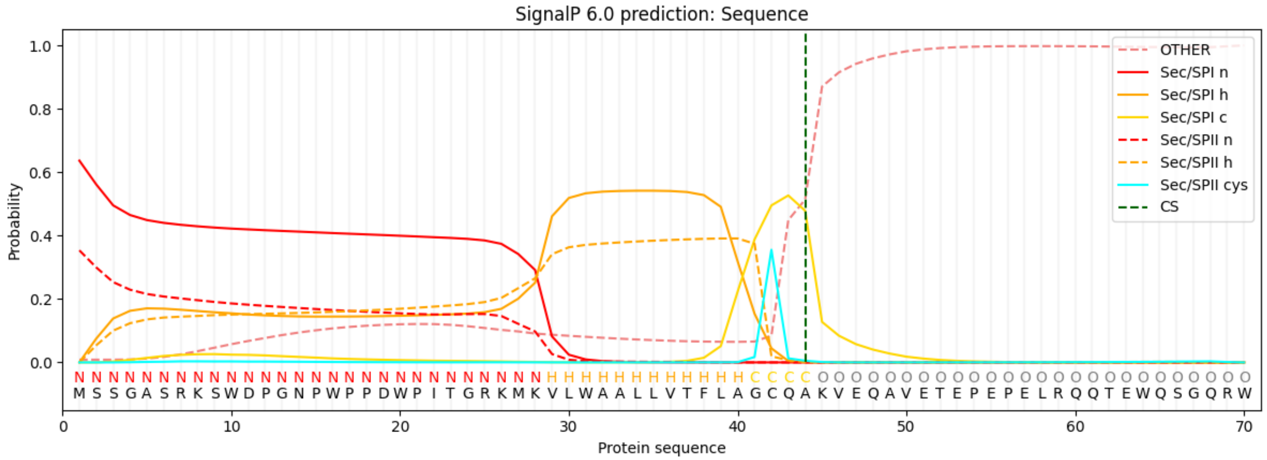

Supplementary Figure S16. Predicted signal peptide of *APOE* transcripts.

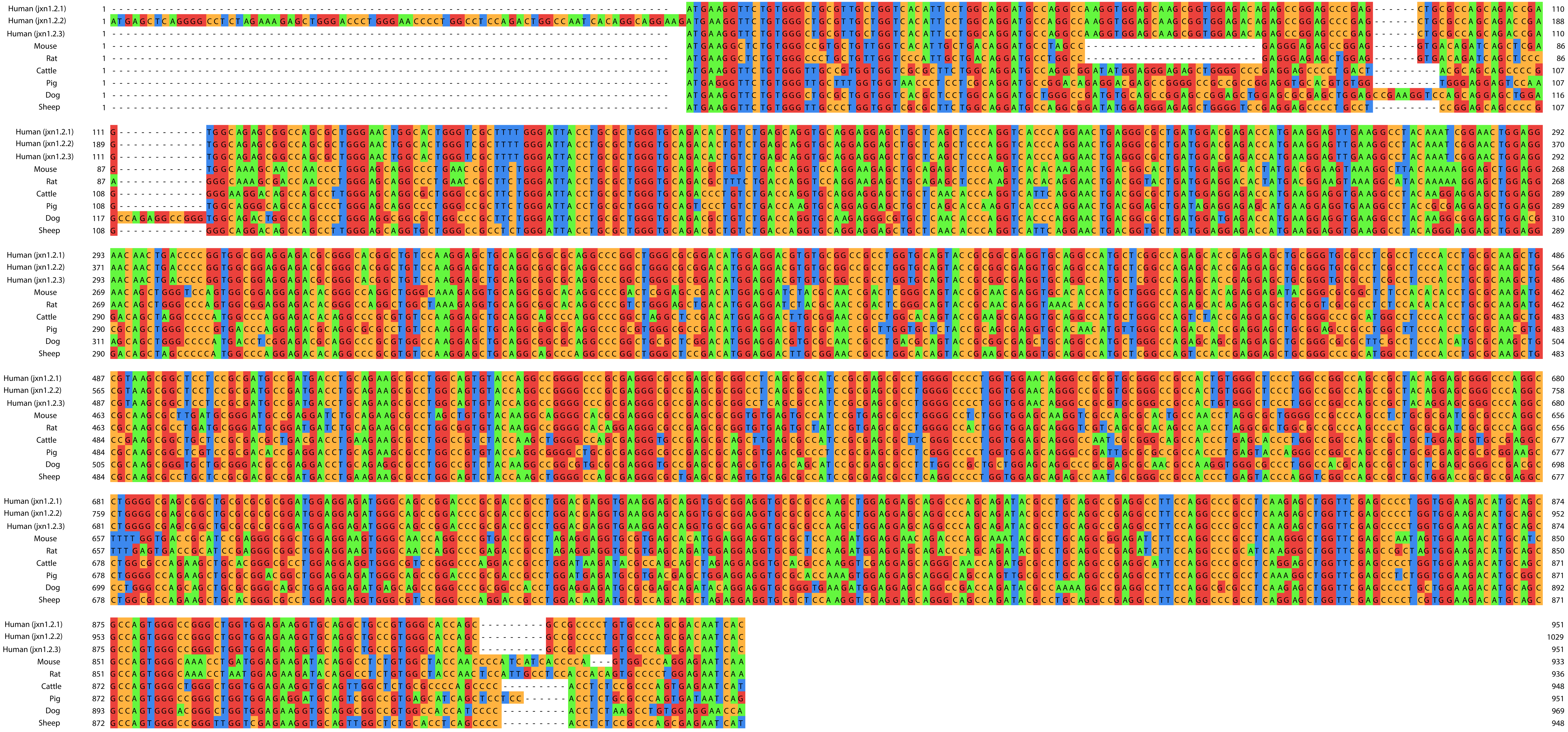

Supplementary Figure S17. Alignment of *APOE* coding sequences across species.

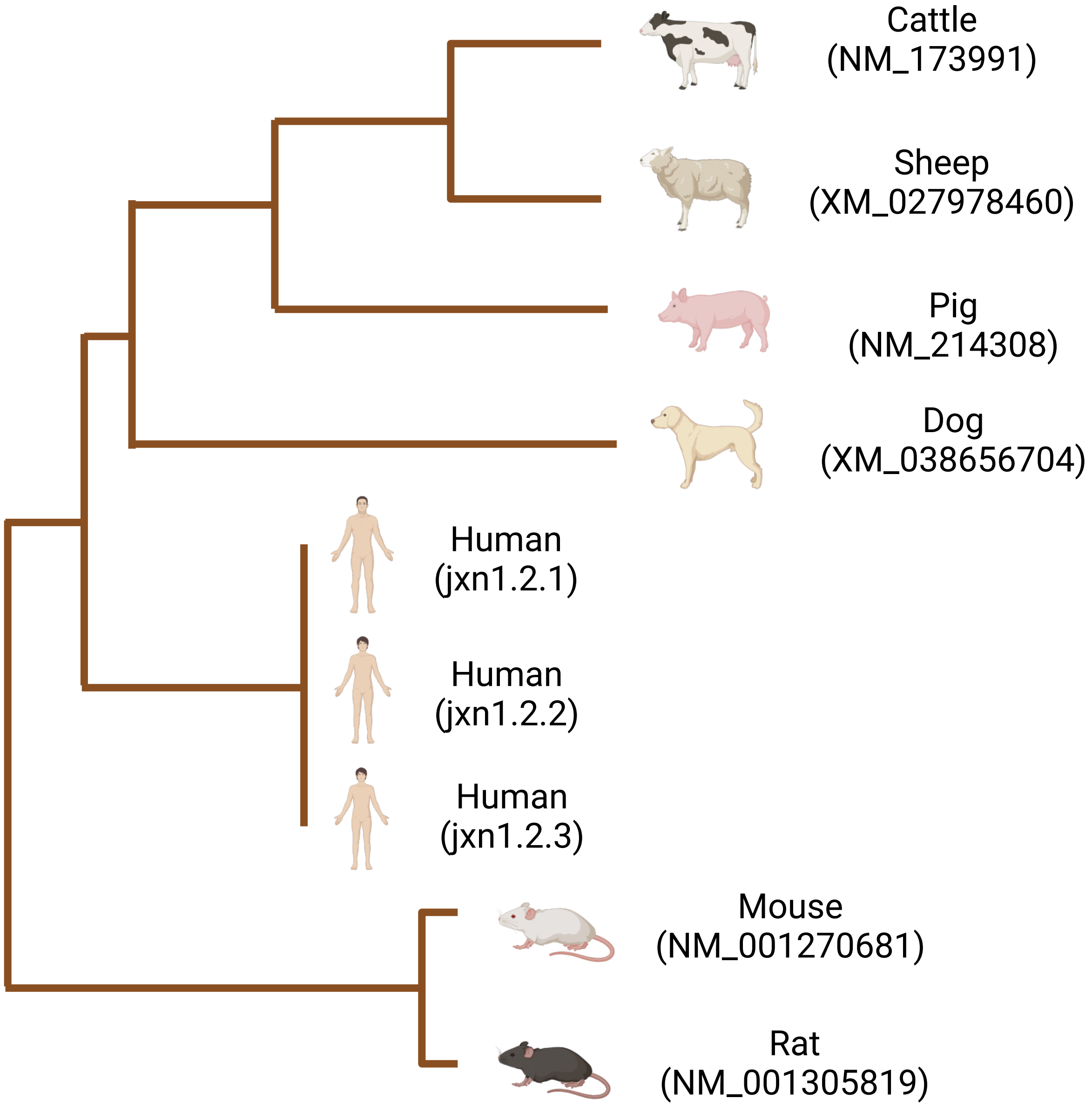

Supplementary Figure S18. *APOE* evolutionary tree across species.

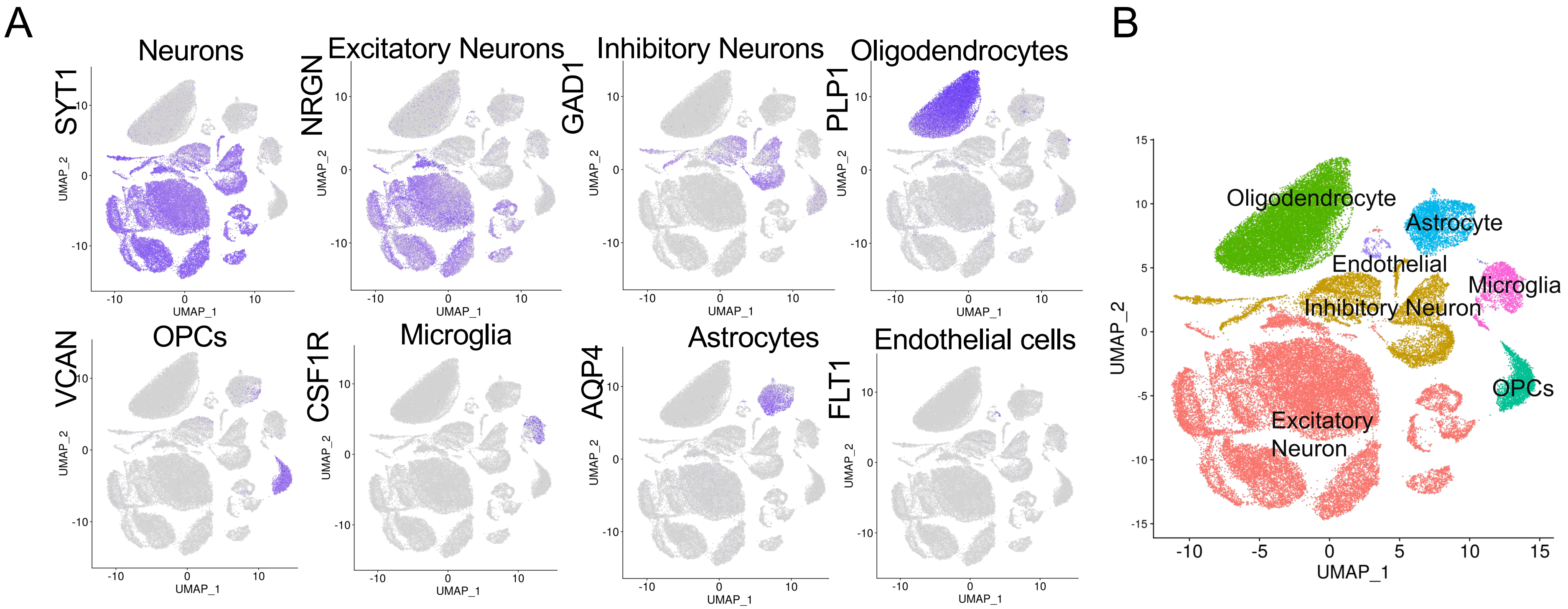

Supplementary Figure S19. Single nucleus sequencing of human brains recapitulates cell-type-specific marker genes (A) and cell types (B).

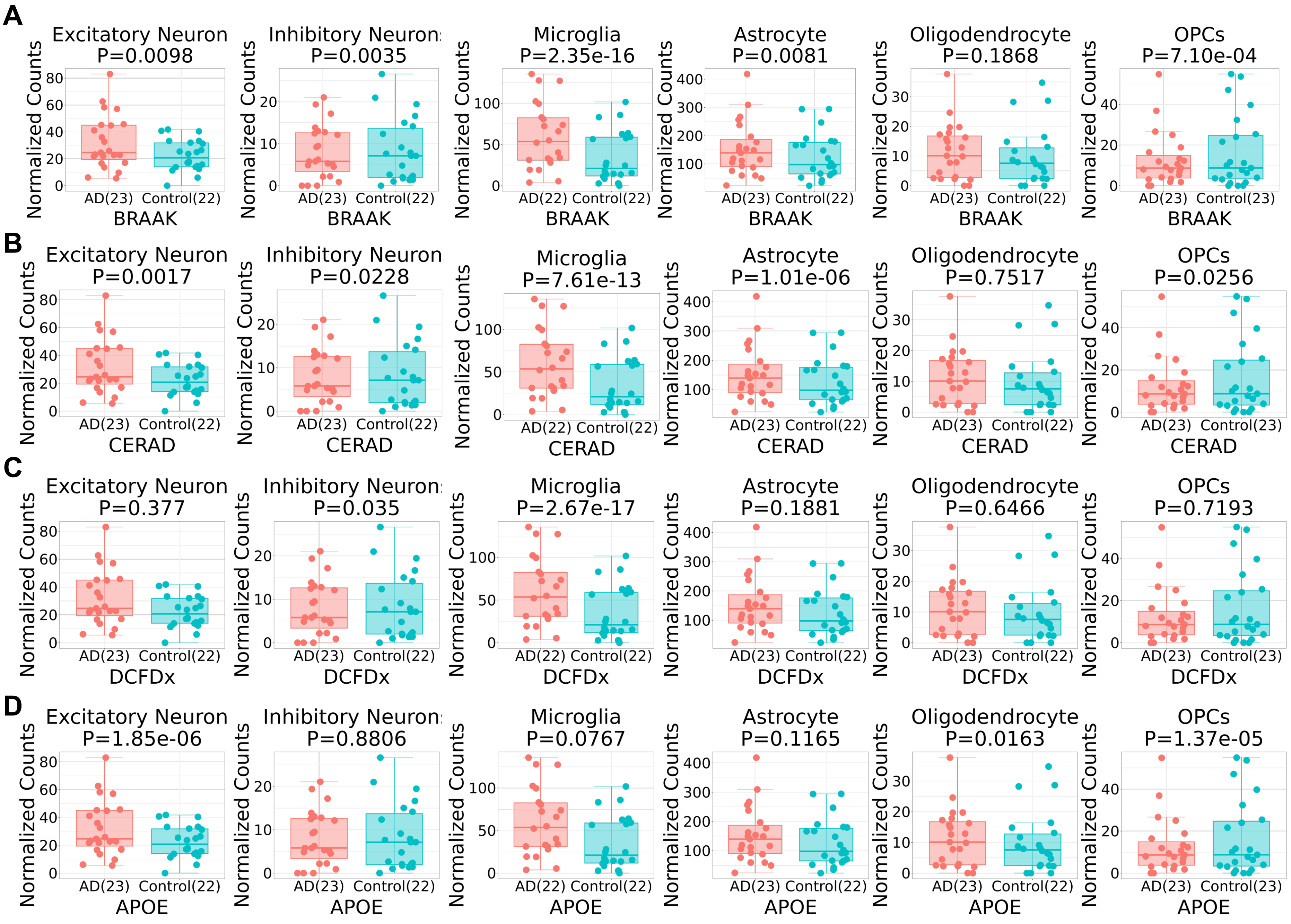

Supplementary Figure S20. Differential expression of *APOE* across 6 cell types in DLPFC between AD and healthy controls in (A) braak stages, (B) CERAD criterion, (C) cognitive impairment, and (D) between *APOE4* carriers vs. non-carriers.

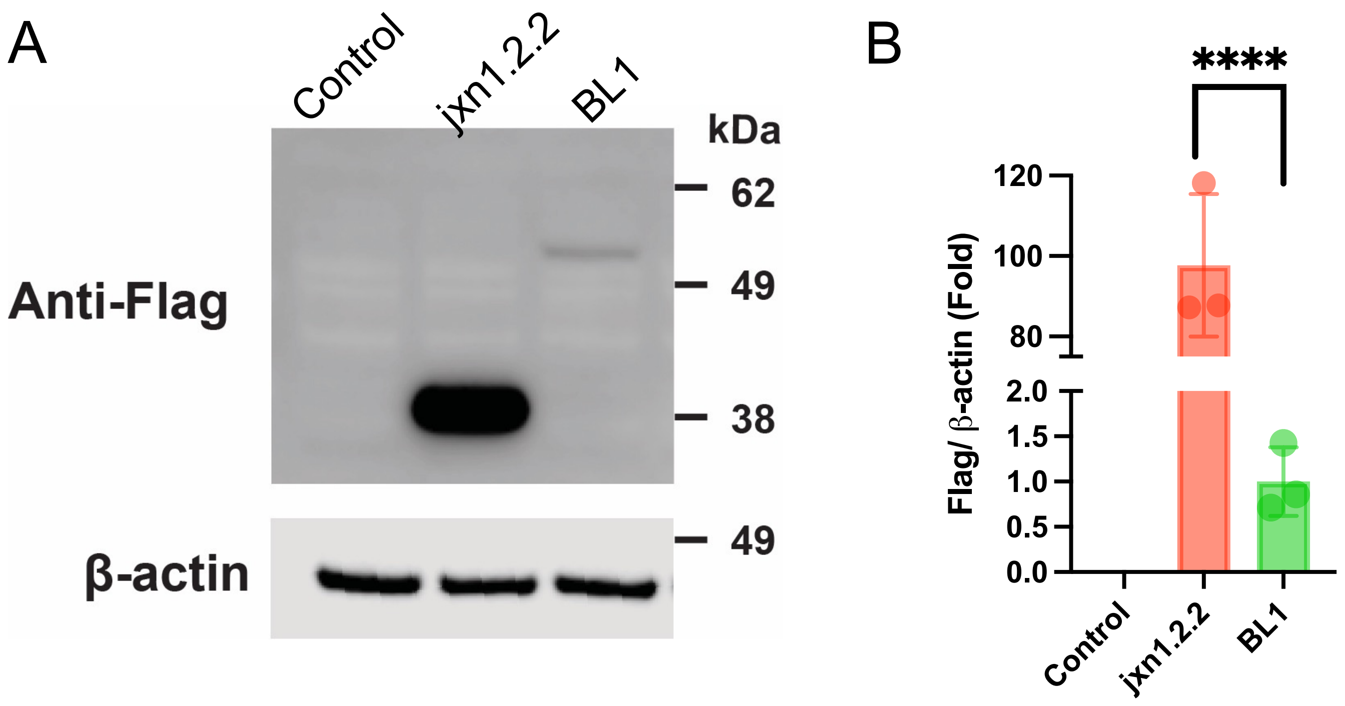

Supplementary Figure S21. The *APOE* jxn1.2.2 transcript is translatable in SK-N-MC cells. (A) Anti-Flag western Blot detected the expression of a Flag-tagged protein in SK-N-MC cells transfected with the APOE jxn1.2.2-Flag construct, as quantified in (B). A Bb1-Flag construct was used as a positive control. The Flag-tagged protein/b-actin ratio in SK-N-MC cells transfected with Bb1-Flag was normalized to 1. Mean ± SEM are shown. One-way ANOVA and Tukey post hoc test, ****p<0.0001.

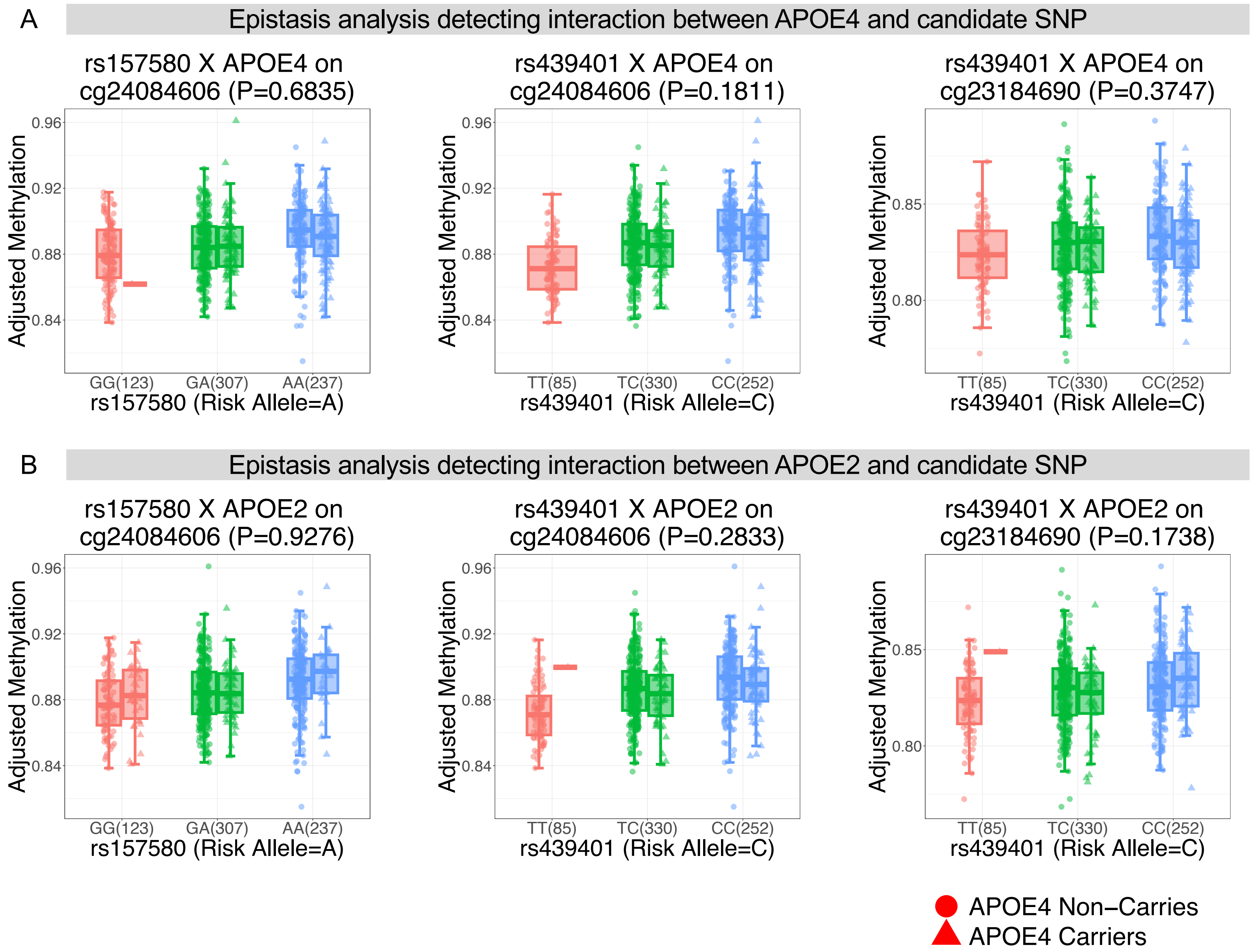

Supplementary Figure S22. Epistasis analysis detects interaction between (A) *APOE4*, (B) *APOE2*, and candidate SNP on DNA methylation levels.

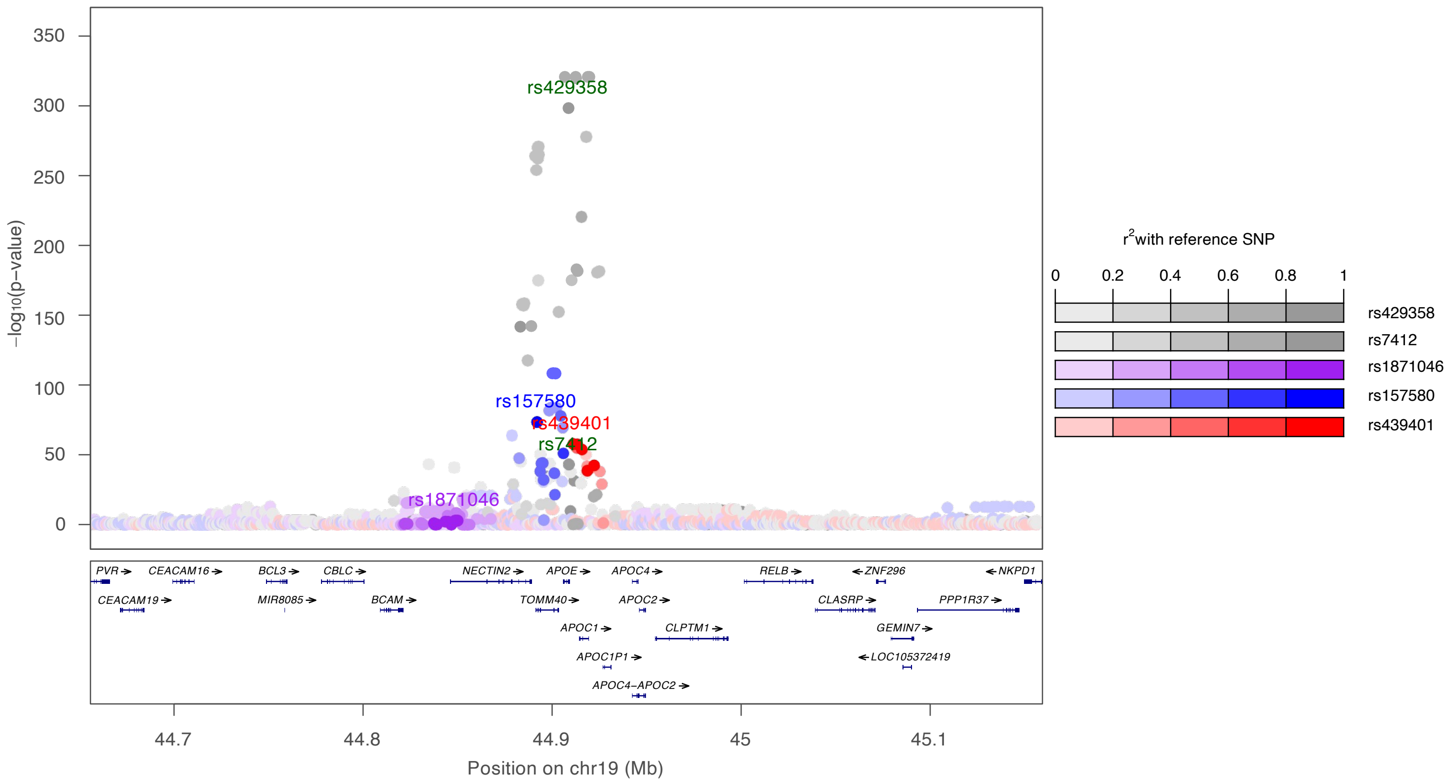

Supplementary Figure S23. A-beta protein GWAS summary statistics at the *APOE* locus (hg38, chr19:44,655,791−45,159,393). Color is coded for linkage disequilibrium of predicted functional SNPs (rs1871046, rs157580, and rs439401) and SNPs consisting of *APOE*2,3,4 genotypes (rs429358 and rs7412).

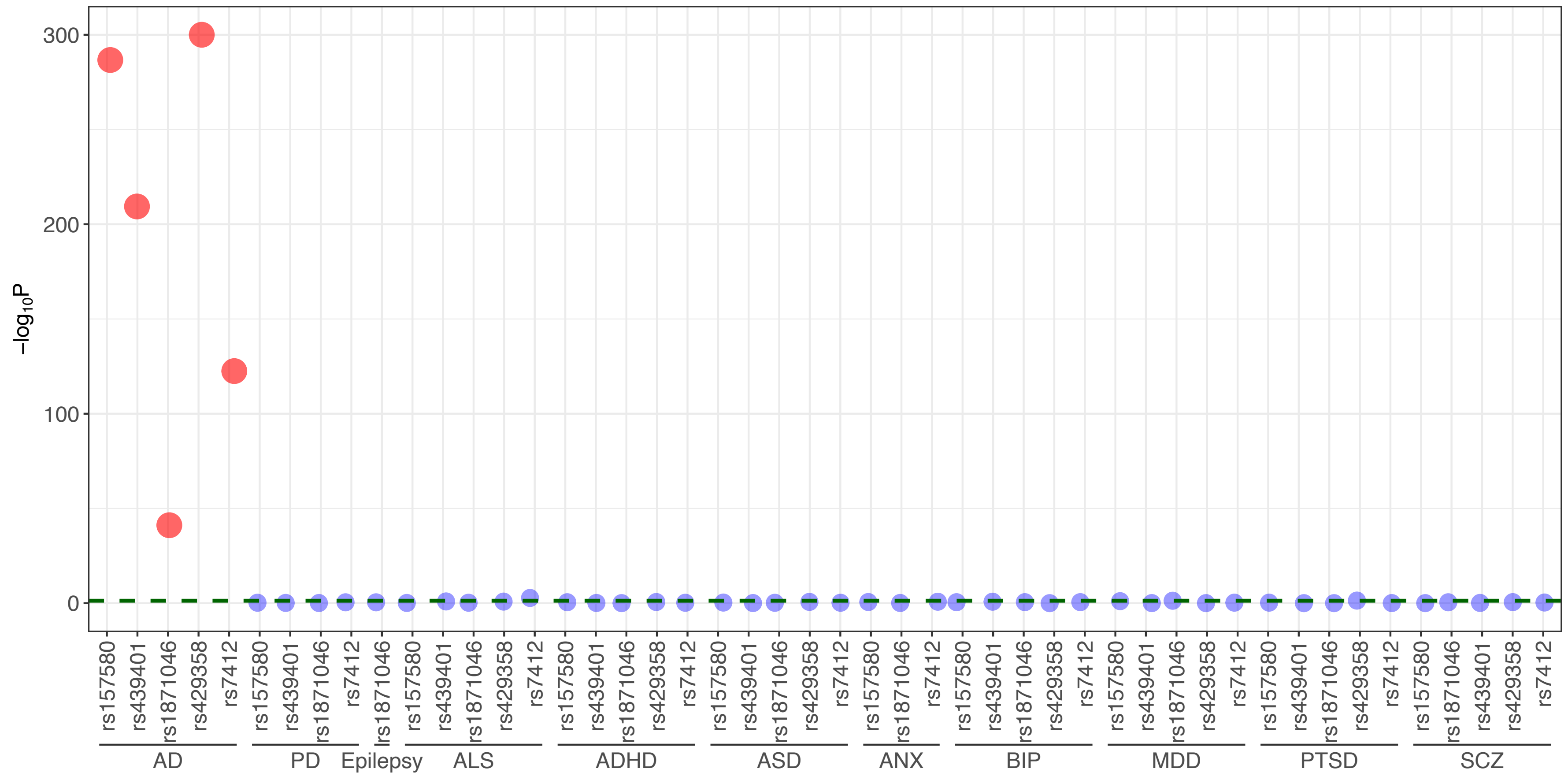

Supplementary Figure S24. *APOE* functional SNPs across GWAS of neurodegenerative and neuropsychiatric disorders. AD, Alzheimer's disease; PD, Parkinson's disease; ALS, Amyotrophic Lateral Sclerosis; ADHD, Attention-deficit/hyperactivity disorder; ASD, Autism Spectrum Disorders; ANX, anxiety disorder; BIP, bipolar disorders; MDD, major depression disorders; PTSD, post-traumatic stress disorder; SCZ, schizophrenia. See GWAS data source in Supplementary File.

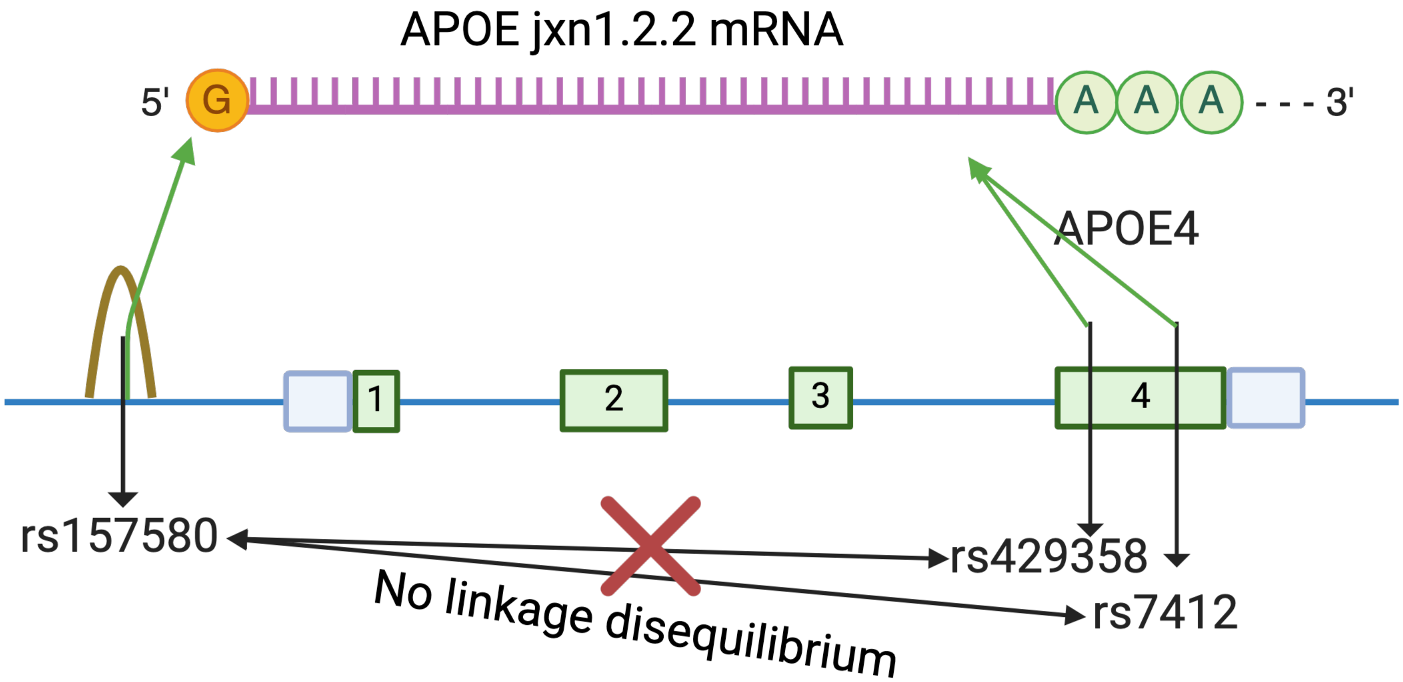

Supplementary Figure 25. The relationship between jxn1.2.2 mRNA, rs157580, and *APOE4*. Note: *APOE4* is derived from SNPs rs429358 and rs7412. See all the *APOE*2,3,4 genotypes in Supplementary Table S3.

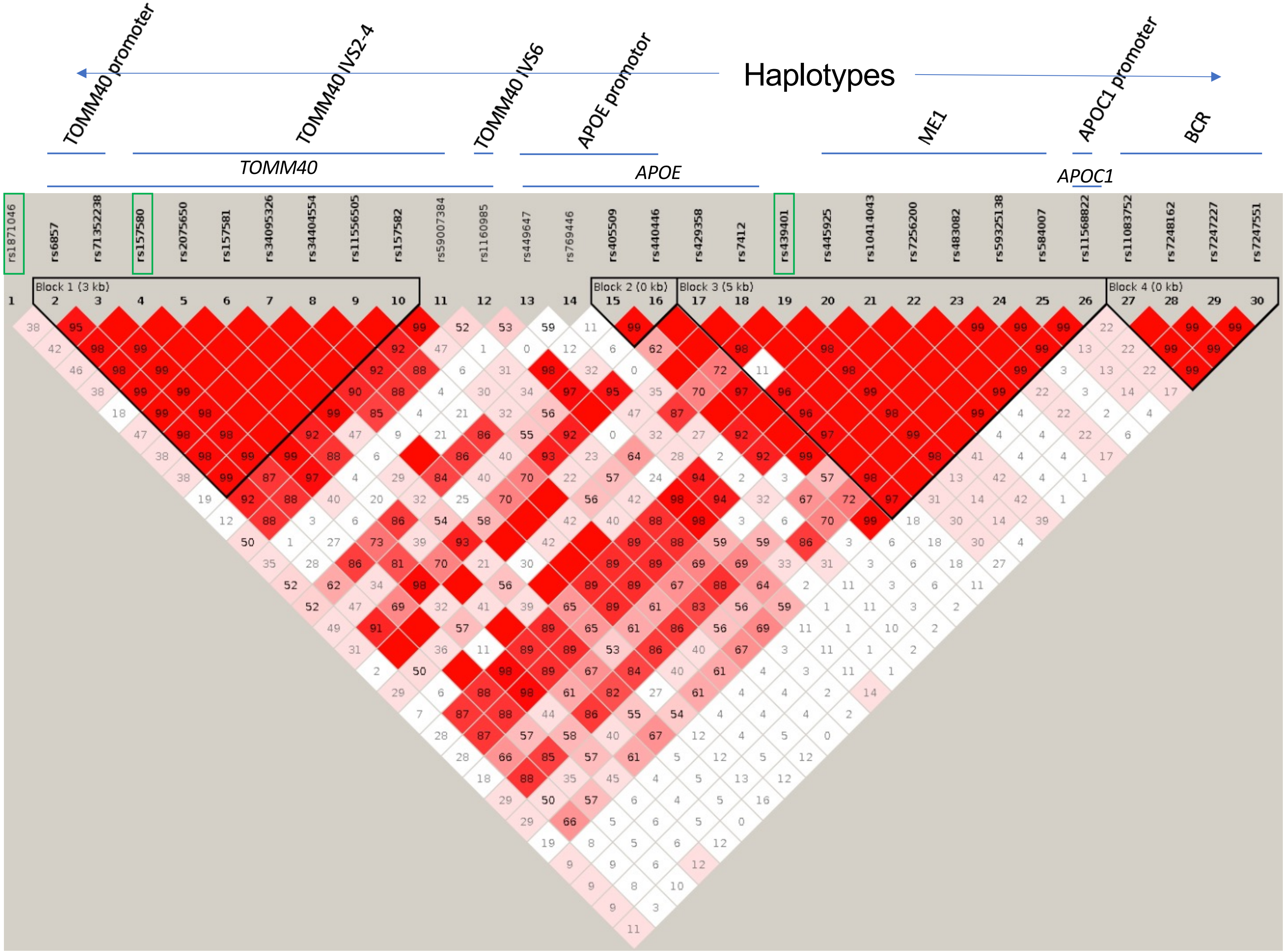

Supplementary Figure S26. *APOE* locus LD plot. *APOE* locus linkage disequilibrium (LD) plot shows the strong LD between *TOMM40* and *APOE*. Higher numbers in squares represent stronger LD calculated using D'. Highlighted regions represent strong haplotype blocks. rs157580 is in the TOMM40 intervening sequence (IVS) 2-4 enhancer region. Plot was generated using ROSMAP European ancestry genotype data with Haploview. Haplotype regions for promotors and enhancers inserted into luciferase reporter constructs were labeled according to *Bekris et al*. (PMID: 22089642).
