# Supplementary material for: Identification of a specific APOE transcript and functional elements associated with Alzheimer’s disease
Source: Mol Neurodegener. 2024 Aug 29;19:63. doi: 10.1186/s13024-024-00751-7 (PMC11361112; doi:10.1186/s13024-024-00751-7)
Supplement: Supplementary file 2 — Supplementary Tables: Table S1. Summary statistics for each RNA-Seq dataset. Supplementary Table S2. Analysis models. Supplementary Table S3. APOE genotypes are derived from two APOE SNPs. Supplementary Table S4. APOE expression trajectory during brain development in BrainSpan data. Supplementary Table S5. Summary statistics of GWAS, eQTL, mQTL across DLPFC, PCC, and AC brain regions at the APOE locus. Supplementary Table S6. Association of candidate SNPs with APOE gene level expression. Supplementary Table S7. Association of candidate SNPs with abundance of specific APOE gene transcripts. Supplementary Table S8. Association of APOE2,3,4 determining SNPs (rs429358 and rs7412) with features including APOE gene expression, APOE transcripts expression, and DNA methylation. Supplementary Table S9. Differential analysis of APOE gene between Alzheimer’s disease and controls across ROSMAP DLPFC, AC, and PCC brain regions; between schizophrenia/bipolar disorders/major depression disorders (MDD) and controls in LIBD Caucasian and African Americans. Supplementary Table S10. Differential analysis of junction between APOE exon 1 and exon 2 between Alzheimer's disease and controls across ROSMAP DLPFC, AC, and PCC brain regions; between schizophrenia/bipolar disorders/major depression disorders (MDD) and controls in LIBD Caucasian and African Americans. Supplementary Table S11. APOE expression trajectory during brain development in LIBD data. Supplementary Table S12. Signal peptide prediction of APOE transcripts. Supplementary Table S13. APOE positive selection LRT and positive selection sites. Supplementary Table S14. Differential expression ofAPOE gene in six major brain cell types across diverse diagnoses. Supplementary Table S15. Transcriptional factors binding to our candidate SNPs. Supplementary Table S16. Summary-based Mendelian randomization (SMR) of rs157580 and rs439401 with APOE jxn1.2.2 transcript. Supplementary Table S17. GWAS summary statistics of candidate SNPs. [file 13024_2024_751_MOESM2_ESM.zip › table.S13.APOE evolution.docx]

Supplementary Table S13. *APOE* positive selection LRT and positive selection sites

| Models | Parameter estimates | LnL | LRT | Positive selection sites |
| --- | --- | --- | --- | --- |
| M7 | $p_{0}=0.45$, $q=1.24$ | -3862.53 | M8 vs. M7  4.34* | 24Q, 28T, 34L, 35R, 272Q, 312G, 318A, 323S |
| M8 | $p_{0}=0.97, p=0.54,$  $q=1.71, p_{1}=0.027,$  $\boldsymbol{\omega=2.48}$ | -3860.36 |  |  |

Sites potentially under positive selection were listed with human APOE first exon and first codon as reference. $\omega$ was the ratio of nonsynonymous-to-synonymous substitution rates. Values of $\omega>1$were highlighted in bold. For the M7 and M8 calculations, $p$ and $q$ were the $\beta$ distribution parameters, and $p_{0}$ and $p_{1}$ were the proportions of amino acids with $0\leq\omega_{0}\leq1$ and $\omega_{1}>1$. LnL log-likelihood; LRT likelihood ratio test.
