# Supplementary material for: Identification of a specific APOE transcript and functional elements associated with Alzheimer’s disease
Source: Mol Neurodegener. 2024 Aug 29;19:63. doi: 10.1186/s13024-024-00751-7 (PMC11361112; doi:10.1186/s13024-024-00751-7)
Supplement: Supplementary file 3 — Supplementary Methods and Results. [file 13024_2024_751_MOESM3_ESM.docx]

# Supplementary Methods

### *APOE* gene evolutionary analysis:

Codeml in PAML software was used to test for the positive selection and infer amino acid sites under the selection^1^. We used likelihood rate test (LRT) for testing positive selection. This was achieved by comparing a null model (M7) that did not allow for sites with $\omega$>1 and a more general model (M8) that did. In model M8 a proportion of sites ($p_{0}$) came from the beta distribution B (p, q) and the remaining sites ($p_{1}=1-p_{0}$) had a $\omega$ ratio estimated from data that can be > 1. Twice the log-likelihood difference was compared with the $\chi^{2}$ distribution value with degree of freedom (*df*) = 2. If the LRT was significant (*P*<0.05), model M8 fit the sequences better than M7, and therefore, the positive selection was inferred.

### Single-nucleus RNA-seq data processing:

Gene counts were obtained by aligning reads using 10x Genomics CellRanger 7.0.0^2^. CellRanger count pipeline was run on each of the 46 individual samples. The CellRanger aggr pipeline was used to aggregate libraries into disease and control groups. The starting dataset contained 68,371 cells. CellRanger count matrices were loaded into the R package Seurat (v4.1.1)^3^. Seurat objects were created to include features detected in at least two cells and to include cells where at least a minimum of 200 features were detected, removing 402 cells. The mitochondrial percentage was calculated using the PercentageFeatureSet function. A high percentage of reads that map to the mitochondrial genome often co-relates to low-quality cells. We removed 4,163 cells with a high ratio of mitochondrial RNA (>30%). We also removed cells with a high total number of molecules detected in them. 25 cells with features/genes greater than 9,000 read counts were removed. Only counts associated with protein-coding genes were considered. After these filtering steps were applied, the dataset included 63,781 cells with 16,847 genes.

Normalization was done with Seurat’s LogNormalize method. With this method, counts for each cell are multiplied by the total counts for all cells, then multiplied by the (default) 10,000 scale factor, and log-transforms the result. We next used the FindVariableFeatures function to calculate highly variable genes per dataset (nFeatures = 3,200). We then applied a linear transformation pre-processing step using the default values in the ScaleData function. PCA was performed on the set of variable genes. Using the FindNeighbors method, we built a k-nearest-neighbor (KNN) graph using the top 50 PC and k = 20 neighbors. FindClusters uses the KNN graph to find clusters using the Louvain (default) algorithm. Once clusters were found, the dataset was projected onto two-dimensional space using Uniform Manifold Approximation and Projection (UMAP) using the RunUMAP function. Seurat identified 20 initial clusters. Using Seurat’s FeaturePlot function to highlight gene expression of known marker genes, as discussed in Mathys et.al.^4^, allowed us to identify and annotate clusters with each of the major brain cell types. Seurat’s FindMarkers function was used to find differentially expressed genes between disease group and control cell types. By default, Seurat adjusted p-value is calculated based on Bonferroni correction. We also performed an FDR by applying R function p.adjust on the p-values.

## GWAS Summary Statistics Source:

| **Label** | **Disorder** | **Journal** | **Year** | **PubMed Link** |
| --- | --- | --- | --- | --- |
| AD | Alzheimer’s Disease | Nature Communication | 2021 | https://pubmed.ncbi.nlm.nih.gov/34099642/ |
| PD | Parkinson’s disease | Nature Genetics | 2023 | https://pubmed.ncbi.nlm.nih.gov/38155330/ |
| Epilepsy | Epilepsy | Nature Genetics | 2023 | https://pubmed.ncbi.nlm.nih.gov/37653029/ |
| ALS | Amyotrophic Lateral Sclerosis | Nature Genetics | 2021 | https://pubmed.ncbi.nlm.nih.gov/34873335/ |
| ADHD | Attention-Deficit Hyperactivity Disorder | Nature Genetics | 2022 | https://pubmed.ncbi.nlm.nih.gov/36702997/ |
| ASD | Autism Spectrum Disorder | Nature Genetics | 2019 | https://pubmed.ncbi.nlm.nih.gov/30804558/ |
| ANX | Anxiety | Molecular Psychiatry | 2019 | https://pubmed.ncbi.nlm.nih.gov/31712720/ |
| BIP | Bipolar Disorder | Nature Genetics | 2021 | https://pubmed.ncbi.nlm.nih.gov/34002096/ |
| MDD | Major Depression Disorder | Nature Genetics | 2019 | https://pubmed.ncbi.nlm.nih.gov/30718901/ |
| PTSD | Posttraumatic Stress Disorder | Nature Communication | 2019 | https://pubmed.ncbi.nlm.nih.gov/31594949/ |
| SCZ | Schizophrenia | Nature | 2022 | https://pubmed.ncbi.nlm.nih.gov/35396580/ |

## CODE AVAILABILITY:

| FastQC | <https://www.bioinformatics.babraham.ac.uk/projects/fastqc/> |
| --- | --- |
| Trimmomatic | <http://www.usadellab.org/cms/?page=trimmomatic> |
| Hisat2 | <https://daehwankimlab.github.io/hisat2/> |
| featureCounts | <https://subread.sourceforge.net/featureCounts.html> |
| MACS2 | <https://hbctraining.github.io/Intro-to-ChIPseq/lessons/05_peak_calling_macs.html> |
| bamCoverage | <https://deeptools.readthedocs.io/en/develop/content/tools/bamCoverage.html> |
| trim_galore | <https://www.bioinformatics.babraham.ac.uk/projects/trim_galore/> |
| bowtie2 | <https://bowtie-bio.sourceforge.net/bowtie2/index.shtml> |
| minifi | <https://bioconductor.org/packages/release/bioc/html/minfi.html> |
| sva | <https://bioconductor.org/packages/release/bioc/html/sva.html> |
| plink | [www.cog-genomics.org/plink/1.9/](http://www.cog-genomics.org/plink/1.9/) |
| TensorQTL | <https://github.com/broadinstitute/tensorqtl> |
| SMR | <https://yanglab.westlake.edu.cn/software/smr/#Overview> |
| Anova | <https://statsandr.com/blog/anova-in-r/#introduction> |
| CellRanger | <https://support.10xgenomics.com/single-cell-gene-expression/software/pipelines/latest/what-is-cell-ranger> |
| Seurat | <https://satijalab.org/seurat/> |
| FIMO | <https://meme-suite.org/meme/doc/fimo.html> |
| regtools | <https://regtools.readthedocs.io/en/latest/> |
| samtools | <https://www.htslib.org/> |
| PSORT2 | <https://psort.hgc.jp/form2.html> |

## Cell culture

SK-N-MC cells (ATCC) were maintained in Dulbecco’s modified eagle medium with high glucose, supplemented with 10% fetal bovine serum and 1% penicillin/streptomycin/amphotericin B (Sigma). Cells were transfected using TransIT-LT1 Transfection Reagent (Mirus Bio) according to the manufacturer’s protocol.

## DNA plasmids

An *APOE* jxn1.2.2-Flag fragment containing the full length jxn1.2.2 (NM_001302688) sequence and a Flag tag to trace the protein encoded by the jxn1.2.2 transcript was gene synthesized by Synbio Technologies (Monmouth Junction, NJ) and subcloned into pcDNA3.1 plasmid with the 5’ UTR downstream of the CMV promoter deleted, so that the transcription starts as the same sites as the endogenous *jxn1.2.2*. A Bb1-Flag plasmid, previously named *FL-7B7D-CAG10*, which expresses a Flag-tagged human Bb1 was as previously described^5^.

## Western blotting analysis

Transfected SK-N-MC cells were lysed with the RIPA lysis buffer supplemented with phenylmethanesulfonylfluoride, sodium orthovanadate, and protease inhibitor cocktail (Santa Cruz Biotechnology). Protein concentrations of lysates were measured using the Pierce™ BCA protein assay kit. Protein samples (30 µg) were loaded to Blot™ mini gel (MES) and gel electrophoresis was run at 200V for 22 min. Gel was then transferred to the novex® nitrocellular membrane with a wet transfer tank at 10V for 60 min. Transferred membrane was blocked with 5% bovine serum albumin (BSA; Sigma-Aldrich) at RT for 1 h. The membrane was then incubated with anti-actin and anti-Flag primary antibodies, respectively, at 4°C overnight. Next day, the membrane was washed with PBS three times with 5min each time. Washed membrane was then probed with HRP-linked secondary antibodies at RT for 2 h. After washing with PBS three times again, the membrane was imaged using the Odyssey Fc Imager (Li-COR Biosciences).

## Statistics

At least three biological replicates of each experiment were performed. Data were presented as mean ± SEM. The results were analyzed using students’ t-test for comparison between two groups, or one-way analysis of variance (ANOVA) followed by Tukey post hoc test for comparison between three or more groups. Statistical significance was set at P value <0.05.

# Supplementary results

### *APOE* underwent positive selection

To investigate whether the *APOE* gene underwent natural selection during evolution, we performed positive selection analysis using a maximum-likelihood method to estimate $\omega$ values among amino acid sites. Positive selection was defined as the presence of some codons at which ω > 1. It indicates that an excess of nonsynonymous over synonymous substitutions was molecular evidence for positive evolution^6^. All the coding sequences of the *APOE* gene were extracted from GenBank (**Supplementary Fig. S17**). A species tree was obtained from the NCBI taxonomy database (**Supplementary Fig. S18**). The $\omega$and log-likelihood values are listed in **Supplementary Table S13**. The LRT indicated that M8 was significantly better fit for the sequences than M7. Under model M8, $\omega$s of 97% sites fit the beta distribution B (0.54, 1.71), and only 2.7% of sites had a $\omega_{1}$=2.48 and were involved in positive selection. The alternative model, M8, was adopted. An excess of nonsynonymous over synonymous substitutions was molecular evidence for positive evolution. Therefore, we demonstrated the presence of positive selection in *APOE*.

Most positive selective genes were involved in sensory perception, immunity and defense^7^. Several studies demonstrated that *APOE* genotypes are associated with stress response-related processes, emphasizing the strong interconnection between mitochondrial function, endoplasmic reticulum stress and the immune response^8-10^. This may be the reason why the *APOE* gene undergoes positive selection. This analysis of molecular evolution helps us to understand *APOE*’s role in immune response from the view of evolution pressure.

## REFERENCES

1. Yang Z. PAML 4: Phylogenetic Analysis by Maximum Likelihood. 2007; *Molecular Biology and Evolution* **24:** 1586-1591.

2. Zheng GX, Terry JM, Belgrader P *et al.* Massively parallel digital transcriptional profiling of single cells. 2017; *Nat Commun* **8:** 14049. PMC5241818

3. Hao Y, Hao S, Andersen-Nissen E *et al.* Integrated analysis of multimodal single-cell data. 2021; *Cell* **184:** 3573-3587 e3529. PMC8238499

4. Mathys H, Davila-Velderrain J, Peng Z *et al.* Single-cell transcriptomic analysis of Alzheimer's disease. 2019; *Nature* **570:** 332-337. PMC6865822

5. Zhou C, Tang F, Dong T *et al.* Role of Bbeta1 overexpression in the pathogenesis of SCA12. 2024; *Mov Disord*.

6. Álvarez-Carretero S, Kapli P, Yang Z. Beginner's Guide on the Use of PAML to Detect Positive Selection. 2023; *Molecular Biology and Evolution* **40:** msad041.

7. Kosiol C, Vinař T, da Fonseca RR *et al.* Patterns of Positive Selection in Six Mammalian Genomes. 2008; *PLOS Genetics* **4:** e1000144.

8. Dose J, Huebbe P, Nebel A *et al.* APOE genotype and stress response - a mini review. 2016; *Lipids in Health and Disease* **15:** 121.

9. Vitek MP, Brown CM, Colton CA. APOE genotype-specific differences in the innate immune response. 2009; *Neurobiology of Aging* **30:** 1350-1360.

10. Maezawa I, Nivison M, Montine KS *et al.* Neurotoxicity from innate immune response is greatest with targeted replacement of E4 allele of apolipoprotein E gene and is mediated by microglial p38MAPK. 2006; *Faseb j* **20:** 797-799.
